# Genome-wide identification of quantitative trait loci for morpho-agronomic and yield-related traits in foxtail millet (*Setaria italica*) across multi-environments

**DOI:** 10.1007/s00438-022-01894-2

**Published:** 2022-04-22

**Authors:** Tianpeng Liu, Jihong He, Kongjun Dong, Xuewen Wang, Lei Zhang, Ruiyu Ren, Sha Huang, Xiaoting Sun, Wanxiang Pan, Wenwen Wang, Peng Yang, Tianyu Yang, Zhengsheng Zhang

**Affiliations:** 1 College of Agronomy and Biotechnology, Southwest University, Chongqing, 400716 China; 2 Crop Research Institute, Gansu Academy of Agricultural Sciences, Lanzhou, 730070 China; 3 Institute of Plant Breeding, Genetics, and Genomics, University of Georgia, Athens, GA 30601 USA; 4 College of Life Science and Technology, Gansu Agricultural University, Lanzhou, 730070 China

**Keywords:** *Setaria italica*, Linkage map, Morpho-agronomic trait, Yield-related traits, QTL

## Abstract

Foxtail millet (*Setaria italica*) is an ideal model of genetic system for functional genomics of the Panicoideae crop. Identification of QTL responsible for morpho-agronomic and yield-related traits facilitates dissection of genetic control and breeding in cereal crops. Here, based on a Yugu1 × Longgu7 RIL population and genome-wide resequencing data, an updated linkage map harboring 2297 bin and 74 SSR markers was constructed, spanning 1315.1 cM with an average distance of 0.56 cM between adjacent markers. A total of 221 QTL for 17 morpho-agronomic and yield-related traits explaining 5.5 ~ 36% of phenotypic variation were identified across multi-environments. Of these, 109 QTL were detected in two to nine environments, including the most stable qLMS6.1 harboring a promising candidate gene *Seita.6G250500*, of which 70 were repeatedly identified in different trials in the same geographic location, suggesting that foxtail millet has more identical genetic modules under the similar ecological environment. One hundred-thirty QTL with overlapping intervals formed 22 QTL clusters. Furthermore, six superior recombinant inbred lines, RIL35, RIL48, RIL77, RIL80, RIL115 and RIL125 with transgressive inheritance and enrichment of favorable alleles in plant height, tiller, panicle morphology and yield related-traits were screened by hierarchical cluster. These identified QTL, QTL clusters and superior lines lay ground for further gene-trait association studies and breeding practice in foxtail millet.

**Supplementary Information:**

The online version contains supplementary material available at 10.1007/s00438-022-01894-2.

## Introduction

Foxtail millet (*Setaria italica*), a member of genus *Setaria*, subtribe Cenchrinae, tribe Paniceae, subfamily Panicoideae, family Poaceae (Kellogg. 2017), has been domesticated and cultivated extensively as a staple food crop for over 11,500 years in China (Wang et al. 2012; Hunt et al. 2021). Foxtail millet has also been developed into a model organism for studying architectural evolution of monocotyledon plant (Doust. 2007; Mauro-Herrera et al. 2016), C_4_ photosynthesis (Brutnell et al. 2010; Mamidi et al. 2020; Chatterjee et al. 2021), nutritional properties (Muthamilarasan et al. 2016), abiotic tolerance and bioenergy in cereal grasses (Kole et al. 2015). In the process of domestication from its ancestor green foxtail (*Setaria viridis*), foxtail millet has undergone a strong artificial selection to generate a wide range of phenotypic changes in branching, plant height, shattering, flowering time and seed size (Li et al. 2021). One of the most critical steps in its domestication was the retention of panicle integrity (i.e. non-shattering) (Schroder et al. 2017; Odonkor et al. 2018). The panicle traits, determining its inflorescence architecture, include primary branch number and density, primary branch length, numbers of branching orders, spikelet number and bristle (sterile branchlet) number, which are also the key morphological characters distinguishing *S. italica* and *S. viridis* (Doust et al. 2002, 2005; Hussin et al. 2020). Identification of QTL and genes underlying these phenotypes is essential to elucidate genetic mechanism of development of *Setaria* and further breeding application with genome editing tools. Then interspecific segregation population helps to mine QTL/genes of phenotypes that have been selected significantly during domestication. From F_2_, F_2:3_ and RIL populations of an interspecific cross between *S. italica* acc. B100 and *S. viridis* acc. A10, QTL for basal branching (tillering), axillary branching, inflorescence architecture related traits, shattering, flowering time, culm height, number of nodes and plant biomass were meticulously detected, and a few genes, such as *tb1*, *SD-1*, *Sh1* and *qSH1*, were identified and evaluated by comparative mapping with other cereal crops (Doust et al. 2004, 2005, 2006; Mauro-Herrera et al. 2013; Odonkor et al. 2018). These QTL and putative genes identified in *S. viridis* served as the basis for foxtail millet domestication.

The existing landraces and contemporary bred cultivars display different panicle types including cylindrical-shaped type, conical-shaped type, spindle-shaped type, and tip-branched type, which could be a result of the variation in primary branch length and density in different parts of the inflorescence. Gene *NEKODE1* responsible for the tip-branched panicle is mapped to the physical position around 13.6–14.4 Mb on chromosome 9 (Masumoto et al. 2016). Furthermore, Hussin et al. (2020) found that a novel member of MADS-box transcription factors, *SiMADS34*, involves in the regulation of panicle width, primary branch length, number of primary branches, panicle length and grain weight. Apart from inflorescence branching, QTL of agronomic and yield-related traits, namely heading data (HD), panicle weight (PW), panicle length (PL), panicle diameter (PD), flag-leaf length (FLL), plant height (PH), stem diameter (SD), stem node number (SNN), code number (CN), code grain number (CGN), thousand-grain weight (TGW), neck length (NL), leaf color (LC), bristle color (BC) and anther color (AC) have been positioned on a high density map derived from different bi-parents intraspecific population (Ni et al. 2017; Fang et al. 2016; Zhang et al. 2017; Wang et al. 2017, 2019b). Among them, QTL for plant height and heading time were elucidated in detail through linkage and bulked segment analyses (BSA) and the relevant genes, such as *Heading date 1 (Hd1)*, *FLAVIN-BINDING, KELCH REPEAT, F-BOX 1(FKF1)*, *Roc4* and *Seita.1G242300*, were predicted by homologous comparisons with close species (Mauro-Herrera et al. 2013; Yoshitsu et al. 2017; Jaiswal et al. 2019; He et al. 2021).

In China, landraces or bred cultivars of foxtail millet has the striking ecotypes or eco-regions that can be classified into the Northeast Plain, North China Plain, Inner Mongolia Plateau and the Northwest Plateau according to the natural climatic conditions of the foxtail millet-producing areas and the heading dates of various groups of foxtail millet varieties (Wang et al. 2012; Jia et al. 2015; Diao et al. 2017). Thus, local adaptation is an important factor in foxtail millet evolution that should be considered in the species breeding programs. *Siprr37* with a transposon insertion was identified as a gene responsible for the adaptation of foxtail millet to the environmental conditions of the early spring sowing region (the Northeast Plain) (Li et al. 2021). Notably, using varieties from different eco-regions to construct a segregation population and dissect quantitative trait loci/gene is necessary and imperative for foxtail millet breeding.

In the present study, we used two cultivars, one from the Northwest Plateau eco-region and the other from the North China Plain eco-region, to cross and construct the RIL population which then was used for genome-wide resequencing and constructing an updated high-density bin map. Seventeen main morpho-agronomic and yield-related traits in breeding practice were phenotyped under four to ten environments. Combined with phenotypes and genotypes, single environment QTL, multi-environment QTL, QTL clusters and six superior lines were identified, revealing the important genomic regions of 17 traits in foxtail millet. These results laid a foundation for fine mapping, identification of candidate genes, elaboration of molecular mechanism of development and breeding application in foxtail millet.

## Materials and methods

### Plant materials

The progenies of the RIL population derived from a cross of Yugu1 and Longgu7, first reported in Liu et al. (2020), were further trialed and phenotyped in 2018, 2019, 2020 and 2021. The F_2:10_ individuals and the parents were grown at Sanya (SY) in the winter of 2018 to the spring of 2019 and two different irrigated fields (DHa: irrigation prior to sowing and at the seedling stage; DHi: irrigation prior to sowing and at the seeding, jointing and filling stages) at DH in 2019, respectively. In 2020, the same sites at DH were used for testing with F_2:11_ lines. For investigating tiller of the RIL population, F_2:12_ were planted in two environments similar to DHa and DHi at Dunhuang and two field (TGh: soil environment with a high phosphorus content; TGl: soil environment with a low phosphorus content) at Taigu (TG) in 2021. The year, irrigation and soil phosphorus conditions in different geographic locations were combined to form 14 test environments (Fig. S1). The geographic location, elevation and other relevant information of the test sites were attached in Fig. S1. All data before 2019 were combined for subsequent analyses.

### Phenotyping

Sequential arrangement method in single factor experimental design was adopted in all test environments and the arrangement order was Yugu1, Longgu7, RIL1, RIL2, …, RIL10, Yugu1, Longgu7, RIL11, RIL12, …, RIL20, Yugu1, Longgu7, … …, Yugu1, Longgu7, RIL141, RIL142, …, RIL164, Yugu1, Longu7 (Fig. S2). In total, 164 RIL lines were used. ~ 80 plants of each seed line were plants. The planting density was 450,000 individuals per hectare. Morpho-agronomic traits were classified into four patterns: 1) stem traits (length of the main stem (LMS), diameter of the main stem (DMS), node number of the main stem (NMS), peduncle length (PL), tiller number (TN)), 2) leaf traits (flag leaf length (FLL) and flag leaf width (FLW)), 3) panicle-related traits (main panicle length (MPL), main panicle diameter (MPD), spikelet density (SD), grain number per spikelet (GNS) and bristle length (BL)), 4) growth period (from emerging to ripen (GP)) and 5) yield-related traits including straw weight per plant (SWP), panicle weight per plant (PWP), grain weight per the main panicle (GWP) and 1000-grain weight (TGW). At the end of filling stage, flag leaf length, flag leaf width and bristle length of 10 plants were measured with a ruler with 1 mm accuracy. After the plant ripening, 15 plants per line were selected randomly and pulled out manually with roots to measure other traits. The diameter of the main stem and main panicle diameter were measured by electronic vernier calipers with accuracy of 0.01 mm. Other length traits were measured with a ruler with accuracy of 0.1 cm. The tiller number, node number of the main stem and spikelet number per panicle were counted visually. Spikelet density was calculated by dividing spikelet number per panicle by main panicle length. After plant drying out, ten spikelets per line were randomly selected to measure grain number per spikelet. The 1000 seeds counted using automatic granule counting machine were weighed to get individual seed weight. Then 15 labeled individual plants were weighed together for straw weight per plant, panicle weight per plant, and grain weight per main stem.

### High-throughput sequencing, sequence alignment and variant calling

The same batch of high-throughput sequencing of the parental lines and 164 F_2:8_ RILs from NCBI under an SRA accession number PRJNA562988 were used as we reported earlier (Liu et al. 2020). More strict analyses were used in this study than previous described (Liu et al. 2020). Briefly, the raw data were filtered by cutadapt (version 1.13) and trimmomatic (version 0.36) software to remove the residual adapter and low-quality sequence (Q score < 30, reads length < 50 bp). Then, the high-quality reads were aligned to the foxtail millet reference genome *S. italica v2.0* (http://plants.ensembl.org/Setaria_italica/Info/Index) using BWA (version 0.7.15-r1140) with MEM algorithm. SamTools software (version 1.3.1) was used to convert the alignment results into BAM format. And SortSAM in Picard tool (version 1.91) was applied to sort the reads in BAM files. Then, using RMDUP in SamTools removed PCR duplication, resulting in a BAM file for further coverage and coverage depth statistics and variant calling. GATK (version 3.7) software package was employed to conduct variant calling for all samples, including SNP and InDel. The resulting variation was further screened according to the following conditions: 1) The proportion of missing genotype in individuals is less than 25%; 2) The frequency of minor alleles is not less than 20%; 3) Observed heterozygosity of individuals is less than 25%.

### Genotyping and construction of linkage maps

Hidden Markov Model (HMM) described by Xie et al. (2010) was adopted to perform genotyping of RIL. Briefly, the parental genotypes were inferred by the linkage relationship between SNP/InDels in the RIL population, and genotypes of each RIL were converted to A and B via inferred genotypes of Yugu1 and Longgu7. Simultaneously, the actual genotypes of Yugu1 and Longgu7 were used to judge the authenticity of the inferring parental genotypes. Based on HMM, the missing genotypes were filled, and the wrong genotypes were corrected. Then the identical SNP and InDel genotypes in the interval were merged to generate bin markers. Furthermore, 74 SSR unevenly distributed on 9 chromosomes of foxtail millet reported previously by Fang et al. (2016) were selected to assess the accuracy of the bin genotype. Then the genetic map distance between markers including bin and SSR was calculated using the Kosambi mapping function in R/onemap (version 2.1.3) and the linkage map was constructed through applying R/LinkageMapview (version 2.1.2).

### QTL identification

MapQTL 6.0 was applied to detect QTL with multiple-QTL models (MQM) mapping. A threshold of log of odds (LOD) ≥ 2.0 indicated the existence of QTL. 1-LOD supporting interval was designated for the confidence intervals (Wang et al. 2019a). The sign “ + ” represented that allele was from Yugu1, while “-” indicated that allele derived from Longgu7. QTL with partially or fully overlapped supporting intervals were regarded as the identical QTL, and QTL detected under two or more test environments was termed as the stable QTL. The letter “q” combining with the trait abbreviation, the chromosome number and the QTL serial number was used to represent the QTL identity.

### Statistical analysis

Descriptive statistics and analysis of variance (ANOVA) were performed for each trait using SPSS Statistics 17.0. Pearson correlation coefficients between traits in different trials were calculated and visualized using R/corrplot package (version 0.90). Package ggplot2 (version 3.3.5) and NbClust (version 3.0) in R language were used to perform the distribution and cluster analysis for all traits, respectively. In the cluster analysis, the function scale was used to standardize the phenotypic values of 17 traits, and then the Nbclust package performed the hierarchical cluster analysis, in which distance and method arguments were euclidean and average, respectively (Charrad et al. 2013). Lem4 package (version 1.1–23) in R language was applied to count heritability for each trait, and the formula is as follows:

$$H = \frac{{V_{g} }}{{V_{g} + \frac{{V_{ge} }}{L} + \frac{{V_{gy} }}{Y} + \frac{{V_{e} }}{LY}}}$$

where *v*_g_, *v*_ge_, *v*_gy_ and *v*_e_ are genetic variance, variance of interaction between genotype and environment, variance of interaction between genotype and year and environmental variance, respectively. L and Y are the number of environments and years, respectively.

## Results

### Phenotypic variation

**Fig. 1:**
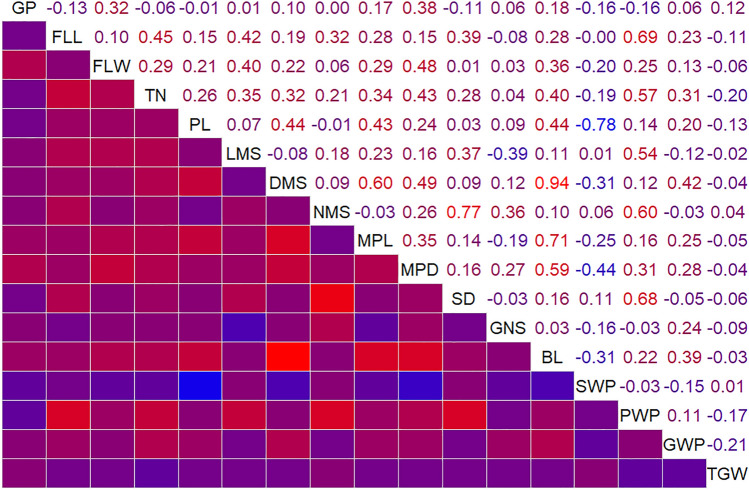
The correlation analysis based on the average of 17 traits at all test environments

All traits were classified into five categories based on plant different organs and growth habit, namely stem, leaf, panicle, growth period and yield-related traits. Among stem-related traits, LMS, DMS, NMS, PL and TN were measured separately in 10, 9, 9, 8 and 8 testing environments, respectively. The values of these traits in general increased with the increase of irrigation and growth period. Almost all the traits conformed to the normal distribution and were typical quantitative traits (Table S1, Fig. S3). However, growth period and tiller number appeared more different under all tested environments. Growth period was longer than other trails in HN, and displayed two peaks under DHa and DHi in 2020, suggesting that it may be regulated by multiple major-minor effect loci. Mean of tiller number was higher at WW, SY and TG than other sites. Four yield-related traits were greater at HN and DH than other tested trials. Panicle weight per plant and grain weight per plant were lowest at SY than other places. On stem related traits, length of the main stem and nodes of the main stem were greater at WW than other environments. All traits are significantly affected by environment factors and a significant (*P* < *0.01*) interaction between genotype and environment (Table S2). But the relationships among all traits were significantly different. There were significantly positive relationships between yield-related traits and panicle-related traits, such as panicle length and the main panicle diameter, and a common negative relationship between grain number of spikelet and spikelet density on all tested environments (Fig. 1, Fig. S4). Only flag leaf length and flag leaf width were measured for leaf-related traits in nine of the fourteen tested environments. Both traits were significant positively and higher at DHi in 2019 and 2020. Among all traits, heritability of MPD, LMS and SWP was higher than 0.9. GP, FLL, PL, MPL and SD were between 0.8 and 0.9. The range of FLW, DMS, NMS, GNS, PWP and TGW was 0.7 to 0.8. BL and GWP were lower than 0.7. The heritability of TN was the lowest (Table S3).

### Sequencing, SNP/InDel identification, and bin map construction

**Fig. 2:**
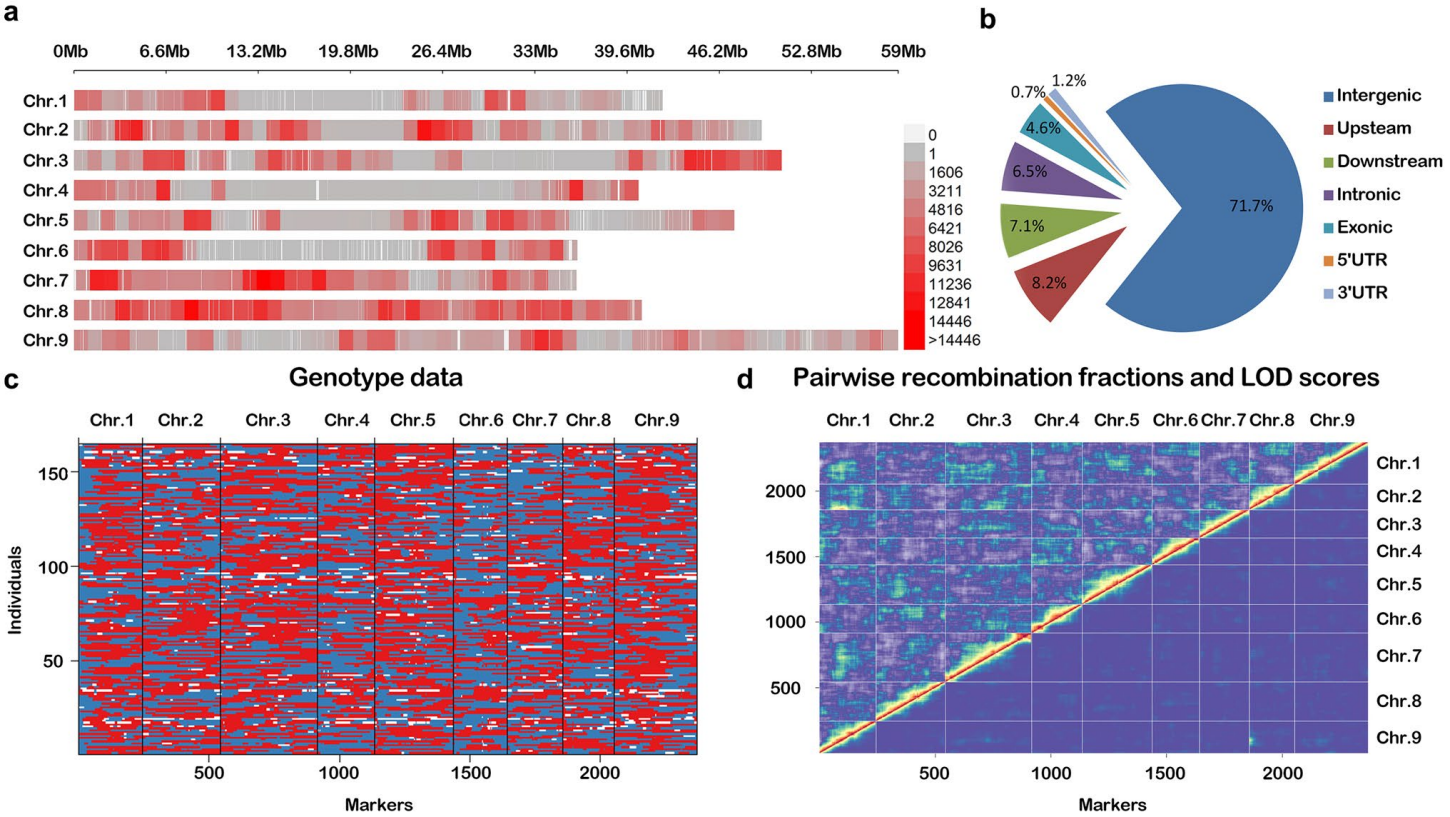
SNP/InDels variations distribution and bin map on Yugu1 × Longgu7 RIL population. **a** SNPs/InDels density map on nine chromosomes; **b** SNP/InDels variations distributed on the genome-wide; **c** The map harboring 2099 bin and 74 SSR markers in the RIL population. **d** The heatmap for genotypic data checking by pairwise recombination fractions (upper left) and logarithm (base 10) of odds (LOD) scores. Red corresponds to complete linkage markers, whereas blue indicates markers with reasonable order and pairwise recombination fractions

Resequencing for 164 F_2:8_ lines and two parental lines was completed previously (Liu et al. 2020). After filtering, there was a total of 1,929,632,243 clean reads with average of 11,624,291 reads per line (Table S4). The 87.8% of the reads were uniquely mapped to the reference genome, with an average 7.45 × depth and 92.8% genome coverage for each line (Table S4). In total, 1,325,599 SNPs and 167,818 InDels were identified from all the samples (Table S5). The number of SNPs and InDels on chromosomes was decreasing in the following order: chromosome 8, 7, 2, 3, 9, 5, 6, 1 and 4, indicating the corresponding order of chromosome genetic differences between two parental lines (Fig. 2a, Table S5). Over 70% of variations were in intergenic regions, and only 11.1% in genic regions. Remaining 15.3% and 1.9% of variations were in upstream/downstream and UTR, respectively (Fig. 2b). The application of the HMM algorithm generated 2,297 bin markers. A bin map from these markers was constructed using R/onemap (Table 1, Fig. 2c). Then, R/LinkageMapveiw was used to construct a linkage map with these bin markers and 74 SSR markers, covering a total of 1315.1 cM on the nine linkage groups of *Setaria*. The interval between adjacent markers ranged from 0.3 to 13.5 cM, with an average interval of 0.56 cM (Table 1, Fig. S5). Analysis of pairwise recombination fractions and LOD scores showed that most of markers had no aberrant phenomenon on the bin map except for few co-segregation markers (Fig. 2d, Fig. S5).

**Table 1:** Marker distribution and genetic distance on nine chromosomes in foxtail millet

Chromosome	Number of SNP	Number of InDel	Number of bin	SSR number	Genetic distance (cM)
Chr.1	87,346	12,717	236	8	128.75
Chr.2	170,948	21,913	293	7	169.7
Chr.3	165,815	21,627	361	11	182.79
Chr.4	76,305	10,703	213	6	117.73
Chr.5	134,675	18,094	289	13	192.42
Chr.6	113,887	15,952	198	7	127.31
Chr.7	177,287	21,011	207	8	125.23
Chr.8	254,692	27,610	188	7	119.21
Chr.9	144,644	18,191	312	7	151.98
Whole	1,325,599	167,818	2,297	74	1315.1

*Chr.*Chromosome, *SNP* Single nucleotide polymorphisms, *InDel* Insertion and deletion

### QTL mapping

A total of 447 loci formed 221 QTL for seventeen traits were detected across multi-environments (Table S6). Of these, 109 QTL were mapped across at least two environments, and 112 QTL were detected on the single environment. The LOD values of these QTL ranged from 2.01 to 15.92, and explained 5.5% to 36% of the phenotypic variations. Among these single environment QTL, 29, 7, 29, 15, 4, 8, 3, 10, and 7 specific QTL were detected separately in 2017DH, 2017HN, 2017WW, 2018GG, 2018HN, 2018SY, 2019DHa, 2019DHi, 2020DHa and 2020DHi, suggesting that there were sharp differences in QTL responding to different environments. The 109 multi-environment QTL mapped in this study were hot genomic regions, which provided valuable information for gene mining and breeding practice of associated traits.

#### QTL for growth period

**Fig. 3:**
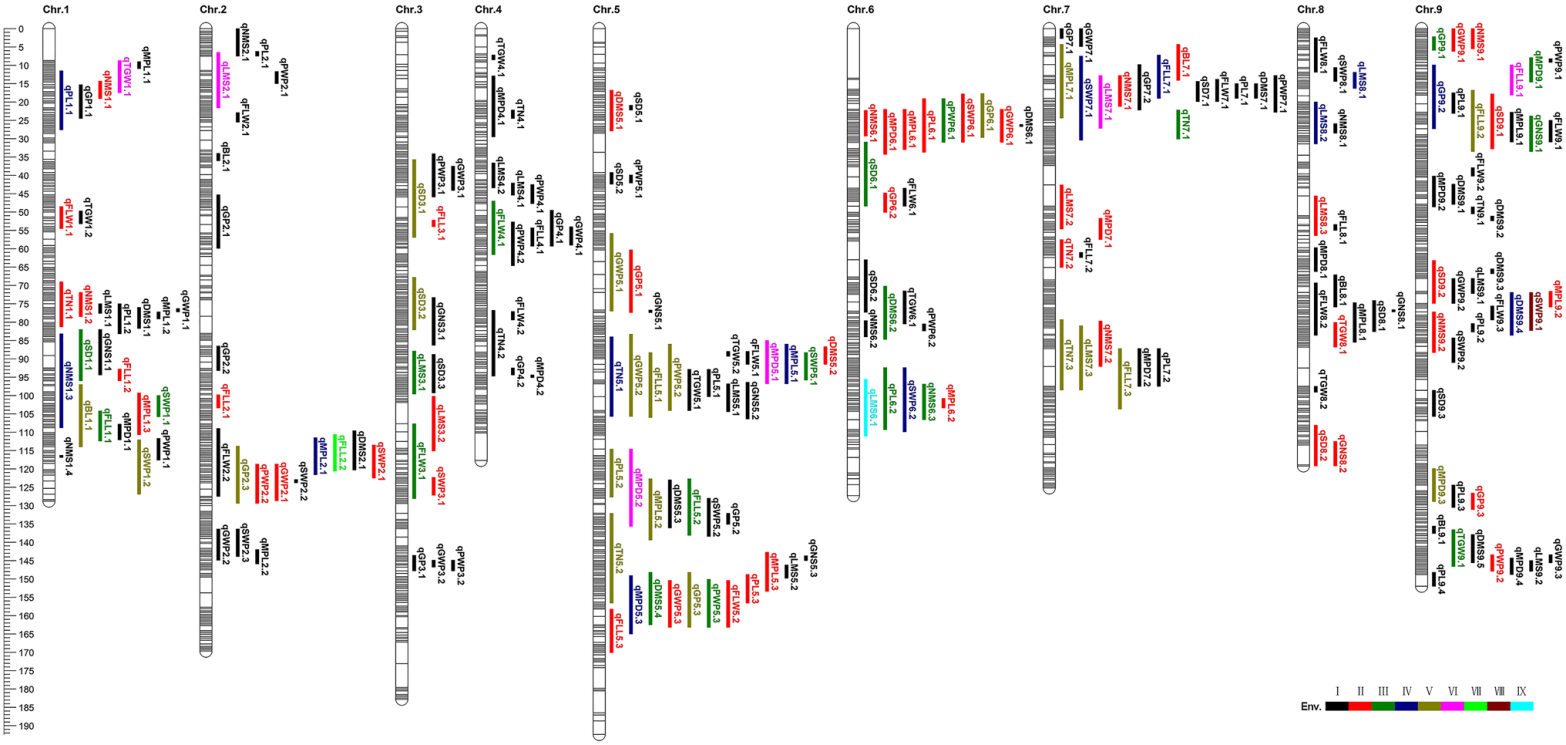
Genetic map and QTL for 17 morpho-agronomic and yield-related traits in the RIL population. The ruler with number on the left indicates the genetic distance in centimorgans (cM). The color intensity on the map represents the marker density. The colors marked by I, II, III, IV, V, VI, VII, VIII, IX represent the one QTL detected on 1, 2, 3, 4, 5, 6, 7, 8 or 9 environments, respectively

Seventeen QTL for growth period (GP) were detected across eight testing environments, distributing on 8 chromosomes except for Chr.8 (Fig. 3). The percentage of phenotypic variance explained by these QTL ranged from 5.5 to 15.8%. The additive effect of the other QTL derived from Yugu1 except for qGP5.1 identified on DHa-2019. Among these QTL, three (qGP2.3, qGP5.3, qGP6.1), one (qGP9.2), one (qGP9.1) and three (qGP5.1, qGP6.2, qGP6.3) were repeatedly identified in five, four, three and two environments, respectively. Nine QTL were specific to only one environment (Fig. 3, Table S6).

#### QTL for flag leaf length

For flag leaf length (FLL), a total of 15 QTL were identified in nine environments and mapped on all chromosomes (Fig. 3), explaining 5.5 to 17.3% of the phenotypic variance. Twelve QTL regions were repeatedly identified in at least two environments, including the only one QTL qFLL2.2 detected in six environments. The Yugu1 alleles had a positive effect for 10 QTL identified except for qFLL1.1, qFLL1.2, qFLL5.3, qFLL7.2 and qFLL7.3 (Table S6).

#### QTL for flag leaf width

For flag leaf width (FLW), 15 QTL were identified in 8 of 9 test environments and mapped on all chromosomes except Chr.2 (Fig. 3), explaining 6 to13.3% of the phenotypic variance. qFLW3.1, qFLW4.1 and qFLW1.1, qFLW5.2 were detected separately in three and two environments. The other QTL were single environment loci. The additive effects of qFLW1.1, qFLW2.1, qFLW3.1, qFLW8.1 and qFLW8.2 originated from Longgu7, while the other QTL were from Yugu1 (Table S6).

#### QTL for tiller number

There were 9 tiller QTL identified at 8 environments, positioning on chromosome 1, 4, 5, 7, 9, and explaining 6–17.6% of the phenotypic variance. Of these, qTN5.2 and qTN7.3 were the two most stable QTL for tiller detected in six environments. qTN5.1 and qTN7.1 were identified in four and three environments, respectively. qTN1.1 and qTN7.2 were the two dual-environments QTL. And the remaining 3 QTL were identified in a single environment. Alleles increasing tiller number were from Yugu1 except for qTN7.1 and qTN9.1 (Fig. 3, Table S6).

#### QTL for peduncle length

Fourteen QTL for peduncle length (PL) were detected in eight environments and distributed on chromosome 1, 2, 5, 6, 7, and 9 (Fig. 3, Table S6). The phenotypic variation explained by these QTL ranged from 5.8 to 18%. qPL5.2, qPL1.1 and qPL6.2 were identified in five, four and three environments, respectively. qPL5.3 and qPL6.1 were detected in two environments. And the remaining 9 QTL were detected under only one environment. Except for qPL1.1, the additive effects for the other QTL were contributed by the Yugu1 (Table S6).

#### QTL for length of the main stem

Seventeen QTL for the length of the main stem (LMS) were detected in ten environments and mapped on all chromosomes, accounting for 5%-36% of phenotypic variance. Among these, ten QTL including the most stable QTL (qLMS6.1) in this study were detected in at least two environments, while the others were environment-specific QTL. The Longgu7 alleles had a positive effect for qLMS1.1, qLMS2.1, qLMS4.1, qLMS4.2, qLMS5.1, qLMS5.2, qLMS6.1 and qLMS9.2, whereas the additive effects for the other QTL were contributed by the Yugu1. (Fig. 3, Table S6).

#### QTL for diameter of the main stem

Fourteen QTL associated with diameter of the main stem (DMS) were detected in six environments and mapped on chromosomes 1, 5, 6, 7 and 9, and explained 5.6–15.1% of the phenotypic variation. Of these QTL, qDMS9.4 was identified across four environments. qDMS5.4 and qDMS6.2, qDMS5.1 and qDMS5.2 were detected three, two environments, respectively. The remaining QTL were identified in only one environment. The additive effect of all the QTL originated from Yugu1, with the exception of qDMS5.1 (Fig. 3, Table S6).

#### QTL for node number of the main stem

Thirteen QTL for node number of the main stem (NMS) were detected in seven environments and located on chromosome 1, 2, 6, 7, 8 and 9 (Fig. 3). The explained phenotypic variation ranged from 5.6 to 22.8%. Among them, qNMS1.3 and qNMS6.3 were detected in four and three environments, respectively. Seven QTL were identified in two environments, and the others were environment-specific QTL. The additive effects for these QTL were contributed by the Yugu1 alleles except qNMS1.1, qNMS1.2, qNMS2.1 and qNMS6.3 (Table S6).

#### QTL for main panicle length

A total of 14 for main panicle length (MPL) were identified in eight environments and they were distributed on all chromosomes except chromosome 3 and 4 (Fig. 3). Among these QTL, two (qMPL5.2, qMPL7.1), two (qMPL2.1, qMPL5.1) and five (qMPL1.3, qMPL5.3, qMPL6.1, qMPL6.2, qMPL9.2) were repeatedly identified in five, four and two environments, respectively, and the remaining 5 QTL for MPL were detected in only one environment. The additive effect of nine QTL was contributed from Yugu1, and the others were from Longgu7 (Table S6).

#### QTL for main panicle diameter

Fourteen QTL for main panicle diameter (MPD) were identified across nine environments and located on all chromosomes except chromosomes 2 and 3, explaining 5.9%–23.7% of phenotypic variance. Of these, qMPD5.1 and qMPD5.2 were detected across six environments, and qMPD9.3, qMPD5.3, qMPD9.1 were detected in five, four and three environments, respectively, which all of the additive effects were from Yugu1. qMPD6.1 and qMPD7.1 were detected in two environments. The additive effect of qMPD6.1 was derived from Yugu1, while qMPD7.1 was from Longgu7. The remaining 7 QTL were identified in a single environment, and all of the additive effects were derived from Yugu1 except qMPD7.2 (Fig. 3, Table S6).

#### QTL for spikelet density

Fourteen QTL for spikelet density (SD) were detected across four environments, and explained 5.5–11.9% of the phenotypic variation of these QTL, two (qSD3.1 and qSD3.2), two (qSD1.1 and qSD6.1), three (qSD8.2, qSD9.1 and qSD9.2) were identified in five, three and two environments, respectively. The favorable allele increasing SD was from Yugu1. The remaining 7 QTL were only identified in a single environment. The additive effects for these QTL were contributed by the Yugu1 alleles, with the exception of qSD7.1 (Fig. 3, Table S6).

#### QTL for grain number per spikelet

Eight QTL for grain number per spikelet (GNS) were detected across three environments and mapped on chromosome 1, 3, 5, 8 and 9, which accounted for 5.8–13.2% of the phenotypic variation. Among them, qGNS8.2 and qGNS9.1 were identified in two environments, while the other six QTL were detected under a single environment. The additive effects of qGNS5.3 and qGNS9.1 were derived from Yugu1 and the other QTL were from Longgu7 (Fig. 3, Table S6).

#### QTL for bristle length

Five QTL for bristle length (BL) were detected in five environments and distributed on chromosome 1, 2, 7, 8 and 9 (Fig. 3). The phenotypic variation explained by the five QTL ranged from 6.3 to 35.2%. qBL1.1 and qBL7.1 were identified in five and two environments, respectively. And the remaining QTL were detected in a single environment. The additive effects of qBL1.1 and qBL7.1 were from Longgu7, while the other QTL were derived from Yugu1. qBL1.1 had the highest percentage of phenotypic variation and was detected in all test environments, which was an important locus to dissect the genetic mechanism of bristle length.

#### QTL for straw weight per plant

Fourteen QTL for straw weight per plant (SWP) were identified in eight environment and mapped on eight chromosomes except for Chr.4, explaining 5.6–14.3% of the phenotypic variation. Of these QTL, three (qSWP2.1, qSWP3.1, qSWP6.1), two (qSWP1.1, qSWP5.1), two (qSWP6.2, qSWP7.1), qSWP1.1 and qSWP9.1 were detected separately in two, three, four, five and eight environments, while the remaining QTL were only detected in a single environment. The favorable alleles of qSWP3.1 and qSWP6.2 were from Longgu7 and the remaining 12 QTL were derived from Yugu1. After adding the test environment, qSWP9.1 were identified for the most stable QTL for SWP (Fig. 3, Table S6).

#### QTL for panicle weight per plant

Fifteen QTL for grain weight per plant (PWP) were detected in nine environments and mapped on the other chromosomes except for chr.8, explaining 5.6–19.5% of the phenotypic variance (Fig. 3, Table S6). Ten QTL were identified in the single environment. qPWP2.2, qPWP9.2 and qPWP5.3, qPWP6.1 were detected in two and three environments, respectively. qPWP5.2 was identified across five environments. The favorable alleles of qPWP2.1 and qPWP2.2 for increasing the trait value came from Longgu7, while favorable alleles of the other QTL were derived from Yugu1. Compared with the published (Liu et al. 2020), qPWP5.2 were identified for a new and more stable QTL controlling panicle weight per plant (Table S6).

#### QTL for grain weight per the main panicle

Fourteen QTL for grain weight per the main panicle (GWP) were identified on eight environments and unevenly distributed on eight chromosomes except for Chr.8, which explained 6.1–15.1% of the phenotypic variance (Fig. 3, Table S6). Of these QTL, eight were detected in the single environment, four (qGWP2.1, qGWP5.3, qGWP6.1 and qGWP9.1) and two (qGWP5.1 and qGWP5.2) were detected in two and five environments, respectively. The favorable alleles of four QTL (qGWP2.1, qGWP2.2, qGWP7.1 and qGWP9.2) came from Longgu7, while the favorable alleles of the remaining QTL were derived from Yugu1. Compared with the published (Liu et al. 2020), after updating the linkage map and adding the test environments, two new stable QTL (qGWP5.1 and qGWP5.2) were mapped on chromosome 5 (Table S6).

#### QTL for 1000-grain weight

Nine QTL for 1000-grain weight (TGW) were detected in seven environments and mapped on chromosomes 1, 4, 5, 6, 8 and 9 (Fig. 3). Of these, 6 QTL were detected in the single environment, while qTGW8.1, qTGW9.1 and qTGW1.1 were identified in two, three and six environments, respectively. The phenotypic variation explained ranged between 5.9 and 8.7%. The positive alleles of the four QTL (qTGW1.1, qTGW4.1, qTGW5.2, and qTGW9.1) originated from Yugu1. The alleles of other five QTL with negative additive effect originated in Longgu7. Compared with the previously published (Liu et al. 2020), after adding the test environments, we identified four new QTL for TGW on chromosomes 1, 5 and 9, of which two QTL (qTGW1.1, qTGW9.1) were the stable loci (Fig. 3, Table S6).

### Stable QTL responsive to multi-environments

**Fig. 4:**
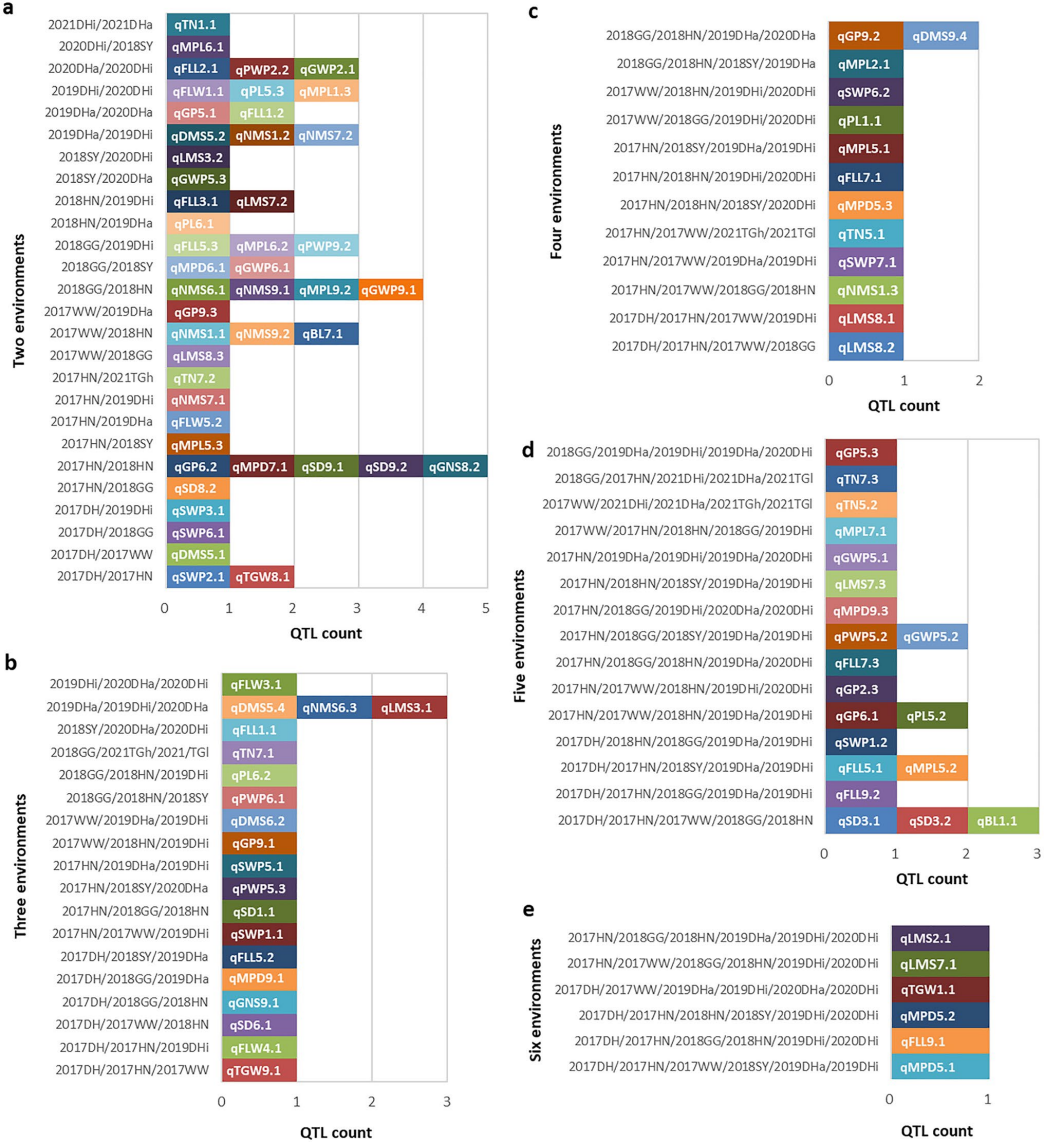
The stable QTL identified in two to six combined environments

One hundred and nine QTL repeatedly detected in two to nine environments were regarded as the stable QTL (Fig. 3, Table S6). Forty-seven QTL were identified in two environments (Fig. 4a). Twenty QTL were identified in three environments (Fig. 4b). Thirteen QTL were identified in four environments (Fig. 4c). Twenty QTL were detected in five environments (Fig. 4d). qLMS2.1, qLMS7.1, qTGW1.1, qMPD5.2, qFLL9.1 and qMPD5.1 were detected in six environments (Fig. 4e). qFFL2.2, qSWP9.1 and qLMS6.1 were identified in seven, eight and nine environments, respectively (Table S6). The test environments 2017HN/2018HN, 2017DH/2019DHa/2019DHi/2020DHa/2020DHi/2021DHa/2021DHi and 2021TGh/2021TGl were located in the same ecologic regions of Huining, Dunhuang and Taigu, respectively, which differed in the year, irrigation and soil conditions. Of the 80 multi-environment combinations formed in 14 test environments, 49 contained at least a pair of test environments with the same ecologic region, in which 70 stable QTL were identified (Fig. 4, Table S6). And among the combination of 2, 3, 4 and 5 environments, 2017HN/2018HN (Fig. 4a), 2019DHa/2019DHi/2020DHa (Fig. 4b), 2018GG/2018HN/2019DHa/2020DHa (Fig. 4c) and 2017DH/2017HN/2017WW/2018GG/2018HN (Fig. 4d) detected the largest number of stable QTL, respectively, suggesting that foxtail millet has more identical genetic modules in the same ecological environment. Four QTL (qGP5.1, qFLL1.2, qGP9.2, qDMS9.4) and eight QTL (qFLL1.1, qPL5.3, qMPL1.3, qSWP6.2, qPL1.1, qFFL7.1, qGP2.3, qLMS7.1) were identified in two years of DHa and DHi environments, respectively (Fig. 4), indicating that these stable QTL were key loci under different irrigation conditions. Furthermore, intervals of the stable QTL were within 0.85–18.41 Mb. The stable QTL, qFLL5.1, qFLL7.1, qFLW3.1, qTN5.1, qSWP5.1, qSWP9.1, qPWP5.2, qGWP5.2, qMPD5.1, qMPL5.1, qMPL7.1 covered over 10 Mb and located in intervals with few variations (Table S6, Fig. 2a). And qGP9.1 carried the smallest interval for 0.85 Mb (Table S6) and covered over 140 genes in foxtail millet annotated genes at Phytozome (https://phytozome.jgi.doe.gov/pz/portal.html#!info?alias=Org_Sitalica).

### QTL clusters for multiple traits

The QTL cluster was defined as a chromosome region harboring multiple QTL for various traits within ~ 20 cM (Tan et al. 2018). And phenotypically correlated traits are often mapped to similar genomic regions (Zhi et al. 2021). Of 221 QTL for seventeen traits, 130 with overlapping intervals formed 22 QTL clusters (Table 2). Chromosome 9 and 5 carried separately five and four QTL clusters and chromosomes 2, 3, 6 and 7 had two QTL clusters, whereas there were three and one clusters on chromosomes 1 and 4, 8, respectively (Table 2). Among these QTL clusters, cluster2.2, cluster3.1, cluster3.2, cluster5.4 and culster9.1 covered individually three QTL. Cluster8.1 and cluster9.3 had separately four QTL. Cluster1.1, cluster1.3, cluster4.1 and cluster6.2 each harbored five QTL. Cluster5.2, cluster7.2, cluster9.2, cluster9.4 and cluster9.5 covered six QTL respectively. Cluster1.2 and cluster5.3 contained individually seven QTL. Cluster2.1 and cluster6.1 each had nine QTL. Cluster7.1 and cluster5.1 harbored 11 and 14 QTL, respectively. Cluster2.2 and cluster3.2 carried QTL mapped only in a single environment. Cluster7.1, cluster5.1, cluster4.1 covered over 10 Mb and mapped in intervals with few variations (Table 2, Fig. 2a). Intervals of QTL cluster were 1.11–22.46 Mb. Moreover, leaf related traits QTL, qFLL2.2 and qFLW2.2, qFLL4.1 and qFLW4.1, qFLL7.1 and qFLW7.1, qFLL9.1 and qFLW9.1 were positioned separately on cluster2.1, cluster4.1, cluster5.1, cluster7.1, cluster9.1 (Table 2). Eleven QTL clusters, cluster1.1, cluster1.2, cluster2.2, cluster5.1, cluster5.3, cluster5.4, cluster6.1, cluster6.2, cluster7.1, cluster7.2, cluster9.4 and cluster9.5, comprised at least two QTL for stem related traits. Nine QTL clusters carried a pair of QTL for panicle related traits. Nineteen out of 22 QTL clusters carried at least one panicle QTL and one QTL for yield related traits (Table 2). These paired traits in the same cluster had significant correlation in the most of test environments (Fig. S4), suggesting there was the existence of pleiotropy or tight linkage in genomic regions of these QTL clusters.

**Table 2:** Information of QTL clusters identified in the RIL population

Chromosome	Cluster	QTL	Physical interval (bp)
Chr.1	Cluster1.1	qMPL1.1^I^, qTGW1.1^VI^, qNMS1.1^II^, qGP1.1^I^, qPL1.1^IV^	45240_5258495
	Cluster1.2	qGWP1.1^I^, qMPL1.2^I^, qDMS1.1^I^, qPL1.2^I^, qLMS1.1^I^, qNMS1.2^II^,qTN1.1^II^	28375053_33874807
	Cluster1.3	qSWP1.1^III^, qMPL1.3^II^, qMPD1.1^I^, qFLL1.1^III^, qBL1.1^V^	35805712_39295447
Chr.2	Cluster2.1	qSWP2.1^II^, qDMS2.1^I^, qFLL2.2^VII^, qMPL2.1^IV^, qSWP2.2^I^, qGWP2.1^II^, qPWP2.2^II^, qGP2.3^V^, qFLW2.2^I^	35201373_41662480
	Cluster2.2	qGWP2.2^I^, qMPL2.2^I^, qSWP2.3^I^	43163245_45068576
Chr.3	Cluster3.1	qGWP3.1^I^, qPWP3.1^I^, qSD3.1^V^	3334993_6785873
	Cluster3.2	qGP3.1^I^, qGWP3.2^I^, qPWP3.2^I^	46589991_47704563
Chr.4	Cluster4.1	qGWP4.1^I^, qGP4.1^I^, qFLL4.1^I^, qPWP4.2^I^, qFLW4.1^III^	5077949_27543440
Chr.5	Cluster5.1	qTN5.1^IV^, qGWP5.2^V^, qFLL5.1^V^, qPWP5.2^V^, qTGW5.1^I^, qPL5.1^I^, qLMS5.1^I^, qGNS5.2^I^, qTGW5.2^I^, qFLW5.1^I^, qMPD5.1^VI^, qMPL5.1^IV^,qSWP5.1^III^, qDMS5.2^II^	11718690_27383654
	Cluster5.2	qGP5.2^I^, qSWP5.2^I^, qFLL5.2^III^, qDMS5.3^I^, qMPL5.2^V^, qMPD5.2^VI^	34633293_38711471
	Cluster5.3	qPL5.3^II^, qFLW5.2^II^, qPWP5.3^III^, qGP5.3^IV^, qGWP5.3^II^, qDMS5.4^I^, qMPD5.3^IV^	41712458_45131176
	Cluster5.4	qLMS5.2^I^, qMPL5.3^II^,qTN5.2^V^	36354717_44234549
Chr.6	Cluster6.1	qDMS6.1^I^, qGWP6.1^II^, qGP6.1^V^, qSWP6.1^II^, qPWP6.1^III^, qPL6.1^II^, qMPL6.1^II^,qMPD6.1^II^, qNMS6.1^II^	1170494_4723612
	Cluster6.2	qMPL6.2^II^, qNMS6.3^III^, qSWP6.2^IV^, qPL6.2^III^, qLMS6.1^IX^	32794293_34859035
Chr.7	Cluster7.1	qPWP7.1^I^, qDMS7.1^I^, qPL7.1^I^, qFLW7.1^I^, qSD7.1^I^, qFLL7.1^IV^, qGP7.2^I^, qNMS7.1^II^, qLMS7.1^VI^, qSWP7.1^IV^, qMPL7.1^IV^	3804624_18883968
	Cluster7.2	qPL7.2^I^, qMPD7.2^I^, qNMS7.2^II^, qTN7.3^II^, qLMS7.3^V^, qFLL7.3^V^	27939599_31644229
Chr.8	Cluster8.1	qGNS8.1^I^, qSD8.1^I^, qMPL8.1^I^, qFLW8.2^I^	31119296_35223660
Chr.9	Cluster9.1	qGWP9.1^II^, qNMS9.1^II^, qGP9.1^III^	15567_2423889
	Cluster9.2	qFLW9.1^I^, qGNS9.1^III^, qMPL9.1^I^, qSD9.1^II^, qFLL9.2^V^, qPL9.1^I^	4491281_ 8,716,958
	Cluster9.3	qPWP9.1^I^, qMPD9.1^III^, qFLL9.1^VI^, qGP9.2^IV^	2771582_5106022
	Cluster9.4	qMPL9.2^II^, qSWP9.1^VIII^, qDMS9.4^IV^, qFLW9.3^I^, qPL9.2^I^, qNMS9.2^II^,	35044370_42223290
	Cluster9.5	qGWP9.3^I^, qLMS9.2^I^, qMPD9.4^I^, qPWP9.2^II^, qDMS9.5^I^, qTGW9.1^III^	55057031_58970233

I, II, III, IV, V, VI, VII, VIII, IX represented the one QTL detected on 1, 2, 3, 4, 5, 6, 7, 8 or 9 environments, respectively

### Superior lines

**Fig. 5:**
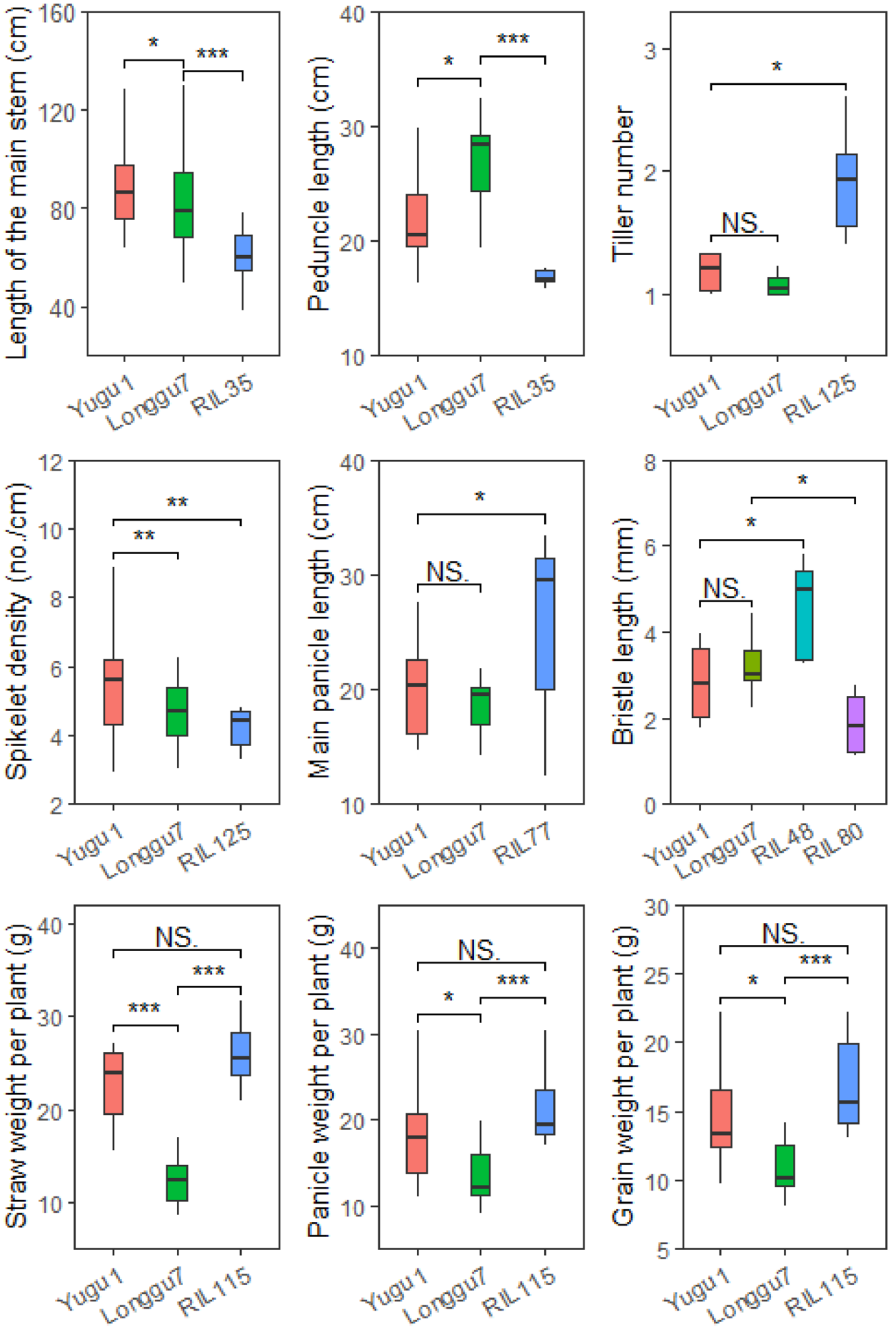
Box plot of over-parent traits of RILs identified by cluster analysis. *, **, *** represent significant differences at *P* < 0.05, 0.01 and 0.001, respectively. NS indicates no significant difference

To select out dwarfing lines and lines with excellent comprehensive characters, we performed cluster analysis with plant height and peduncle length as one kind of variables and the other 15 traits as another kind of variables. Six recombinant inbred lines, including RIL35, RIL48, RIL77, RIL80, RIL115 and RIL125 with transgressive inheritance in plant height and peduncle length, panicle morphology and yield related-traits were identified as superior lines (Fig. S6). Of these, RIL35 was classified into a distinctive branch according to the length of the main stem and peduncle length, both of which had significantly positive correlation in most tested environments (Fig. 1, S4, S6a). Furthermore RIL48, RIL77, RIL80, RIL115 and RIL125 with better comprehensive characters were clustered into another distinctive branch via the remaining 15 phenotypic values (Fig. S6b, Table S7). T-test showed that length of the main stem and peduncle length of RIL35 were significantly shorter than those of parents (Fig. 5). RIL35 carried dwarf genotypes on all 17 QTL regions for length of the main stem and 11 out of 14 QTL regions for peduncle length (Fig. S7a). Similarly, RIL115 having obvious over-parent characteristics in straw weight per plant, panicle weight per plant and grain weight per plant, combined 38 favorable alleles of 43 QTL regions for the three traits (Fig. 5, S7f). RIL125, an over-parent line in spikelet density and tiller, carried 13 of the 14 QTL alleles decreasing spikelet density, 6 of 9 QTL alleles increasing tiller number, respectively (Fig. 5, S7b). RIL77 combined 11 favorable alleles in 14 QTL regions to produce over-parent phenotype in length of main panicle (Fig. 5, S7c). The bristle length of RIL48 and RIL 80 were significantly different from those of parents (Fig. 5). The two lines carried opposite genotypes in the most stable QTL (qBL1.1) and a single environment QTL (qBL8.1) regions, indicating that the two QTL were the most major loci affecting bristle length (Fig. S6 d and e). These results suggested that over-parent characteristics of these traits in superior lines were resulted from an accumulation of favorable alleles.

## Discussion

### An updated linkage map

The previous linkage map was constructed based on F_2_ and RIL population from a cross between Yugu1 and Longgu7 (Fang et al. 2016; Liu et al. 2020). In the present study, we reanalyzed and used a more stringent selection of higher quality SNPs than previously published (Table 1). Xie et al. (2010) developed a parent-independent strategy for genotyping of a mapping population that was considered both parents and population genotypes. Thus, genotype calling of RILs was more accurate in this study than previous. Additionally, we applied 74 polymorphic SSR markers developed by Fang et al. (2016) to further verify the bin map. Pairwise recombination fraction and LOD scores indicated that all markers concord with the linkage inheritance. These results ensure an updated linkage map with a high accuracy and reliability for QTL mapping described here.

### Correlations among traits

Seventeen morpho-agronomic and yield-related traits are the most important in foxtail millet breeding and also the pivotal indicators distinguishing different foxtail millet ecotypes. Interrelationships between them affect foxtail millet morphogenesis and application in practice. QTL and genes for the traits in the same plant organ or those which are intercorrelated are more likely to be located in the same genomic region. In foxtail millet, Zhang et al. (2017) positioned the QTL for PL, FLL, and PH that all clustered on the chromosomes 5 and 9. Zhi et al. (2021) identified 34 co-located QTL clusters involving nine traits related to panicle architecture and grain yield. Wang et al. (2019b) suggested that intercorrelated traits were probably associated with the growth module and genetic regulation pathway. In this present study, we identified a similar trend and correlation, for example, with cluster1.2, cluster 1.3, cluster2.1, cluster5.3, and cluster9.4 covering multiple QTL for LMS, MPL, PL and BL. Similarly, measures of width (DMS, DMP and FLW) and measures of weight (SWP, PWP, GWP and TGW) were intercorrelated. Furthermore, QTL for some negatively correlated traits, such as qSD9.1 and qGNS9.1, belonged to the same cluster 9.2. These results indicate that there was a presence of genetic linkage or pleiotropy effects among traits with significant correlation (Fang et al. 2016; Zhi et al. 2021).

### Favorable QTL alleles and superior lines

The favorable alleles for a trait do not necessarily come from the more favorable parent (Wang et al. 2019a). In foxtail millet QTL mapping, Wang et al. (2017) identified that the additive effects of 7 out of the 11 major QTL for plant height, main panicle length, main panicle diameter, first main internode diameter, second main internode diameter, and third main internode diameter and main panicle weight per plant were from inferior parent. Among the 26 QTL for plant height identified by He et al. (2021), the dwarfing favorable alleles of 8 QTL came from the parent with higher plant height. Zhi et al. (2021) identified 159 QTL for panicle architecture and grain yield-related traits, of which the additive effects of 60 QTL were from Ai88 and the additive effects of 89 QTL were from Liaogu1. In the present study, the 74 and 147 QTL alleles increasing phenotypic values originated from Longgu7 and Yugu1, respectively. These results indicated that both the superior and inferior parent could contribute QTL alleles that increase the trait values. The accumulation of parental favorable alleles leads to the generation of over-parent lines in progeny populations (Wang et al. 2019a). Kulkarni et al. (2020) identified that six RILs possessing the major yield related QTL and fertility restorer loci *Rf3* and *Rf4* alleles were complete restorers, which can be useful in hybrid rice breeding. Wang et al. (2020) identified that phenotypically superior RIL47 with the major QTL genotypes linked to rice photosynthesis-related traits (PRT) could be considered for genetic improvement of PRT under cold water stress. In this study, six superior recombinant inbred lines, RIL35, RIL48, RIL77, RIL80, RIL115 and RIL125 combining multiple favorable alleles from bi-parents and generating transgressive inheritance in plant height, tiller, panicle morphology and yield related-traits were identified for superior lines, which could be applied into foxtail millet breeding programmes.

### The necessity of fine mapping for the stable QTL

One of the main aims of QTL mapping is to identify and clone causal genes affecting target traits (Liu et al. 2012). QTL mapping is affected by the resolution of genetic map, the size of the population, and the accuracy of the phenotyping. The construction of high density or ultra-high density improved the precision of QTL mapping in foxtail millet (Zhang et al. 2017; Wang et al. 2019b; He et al. 2021; Tian et al. 2021). However, the amount of variations between parental lines and the degree of uniform distribution on chromosomes further affects the resolution of the map. Therefore, chromosome segments with dense markers and small haplotype blocks are preferred for mapping QTL into relatively small intervals. Previously, based on the 333 RIL population and 3744 bin markers, He et al. (2021) mapped and predicted a plant-height QTL gene (QTG), *Seita.1G242300* in the interval with dense variations through homologous comparison with relative species. In this study, utilization of high-density markers improved the resolution of mapping. However, qGP9.1 with the smallest interval on the terminal of chromosome 9 still harbored over 140 genes, so it is difficult to map and clone quantitative trait genes on the primary population with a small number of individuals or intervals with fewer variations. Fang et al. (2017) established an F_2_ secondary population with 2484 individual plants from a cross between recombinant line RIL014 and CCRI35 and fine mapped qFS07.1 into a 62.6 kb genome region containing four annotated genes, which greatly improved the precision of candidate gene identification. Then, fine mapping of qGP1.1 or all stable QTL will be necessary through constructing secondary population with the larger number of individuals. Moreover, qLMS6.1, a most stable QTL within 33,367,330–34,859,035 on chromosome 6, was mapped across nine environments (Table S6, Fig. 3). The QTL for plant height was also identified in fewer environments by Jia et al. (2013), Fang et al. (2016), Zhang et al. (2017) and He et al. (2021). *Seita.6G250500* underlying qLMS6.1, an ortholog to *Os08g44590* in rice, is a putative candidate gene, involving in GA biosynthesis or signaling pathways (He et al. 2021). Similarly, the qLMS6.1 region contains 188 annotated genes (https://phytozome.jgi.doe.gov/pz/portal.html#!info?alias=Org_Sitalica). whether *Seita.6G250500* is the major effect gene is still uncertain, so fine mapping of the QTL will be necessary for the precise identification of causal gene and functional verification.

## Conclusion

An updated high-density genetic map harboring 2297 bin and 74 SSR markers was constructed, which covered 1315.1 cM with an average distance of 0.56 cM between consecutive markers. Two hundred twenty-one QTL including 103 stable QTL and 22 QTL clusters for 17 morpho-agronomic and yield-related traits were identified across 14 environments. Six recombinant inbred lines were identified as superior materials in plant height, tiller, panicle morphology and yield related-traits. This study provided insights into the genetic dissection of 17 morpho-agronomic and yield-related traits in foxtail millet.

## Supplementary Information

Below is the link to the electronic supplementary material.

Supplementary file1 (PNG 746 KB)

Supplementary file2 (PDF 51 KB)

Supplementary file3 (PNG 75 KB)

Supplementary file4 (PDF 891 KB)

Supplementary file5 (PDF 108 KB)

Supplementary file6 (PNG 73 KB)

Supplementary file7 (PDF 347 KB)

Supplementary file8 (DOCX 31 KB)

Supplementary file9 (DOCX 17 KB)

Supplementary file10 (DOCX 16 KB)

Supplementary file11 (XLSX 25 KB)

Supplementary file12 (DOCX 15 KB)

Supplementary file13 (XLSX 47 KB)

Supplementary file14 (DOCX 18 KB)

## Data Availability

Raw sequencing data of parental and recombinant inbred lines has been deposited at NCBI under an SRA accession number PRJNA562988 and can be accessed through the link https://www.ncbi.nlm.nih.gov/bioproject/PRJNA562988. SSR primers used in present study have been deposited in the probe databases of NCBI in http://www.ncbi.nlm.nih.gov/probe/?term=JAK%5Bsubm%5D%20. The other data sets supporting the results of the article are included within the article and its supplementary information files.
