# Supplementary figures and images for: Genome-wide identification of quantitative trait loci for morpho-agronomic and yield-related traits in foxtail millet (Setaria italica) across multi-environments
Source: Mol Genet Genomics. 2022 Apr 22;297(3):873–88. doi: 10.1007/s00438-022-01894-2 (PMC9130181; doi:10.1007/s00438-022-01894-2)

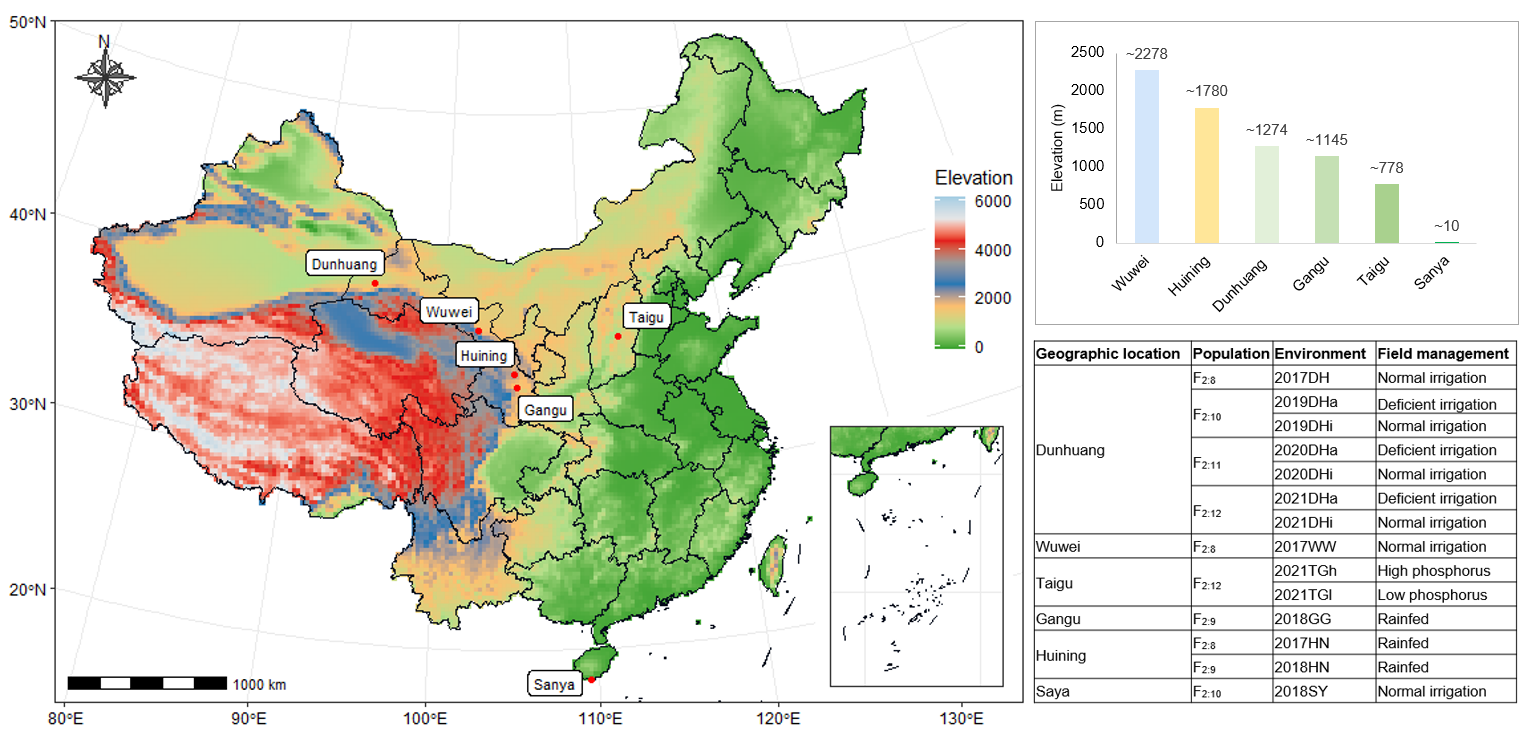

Supplement: Supplementary file 1 — Supplementary file1 (PNG 746 KB) [file 438_2022_1894_MOESM1_ESM.png]

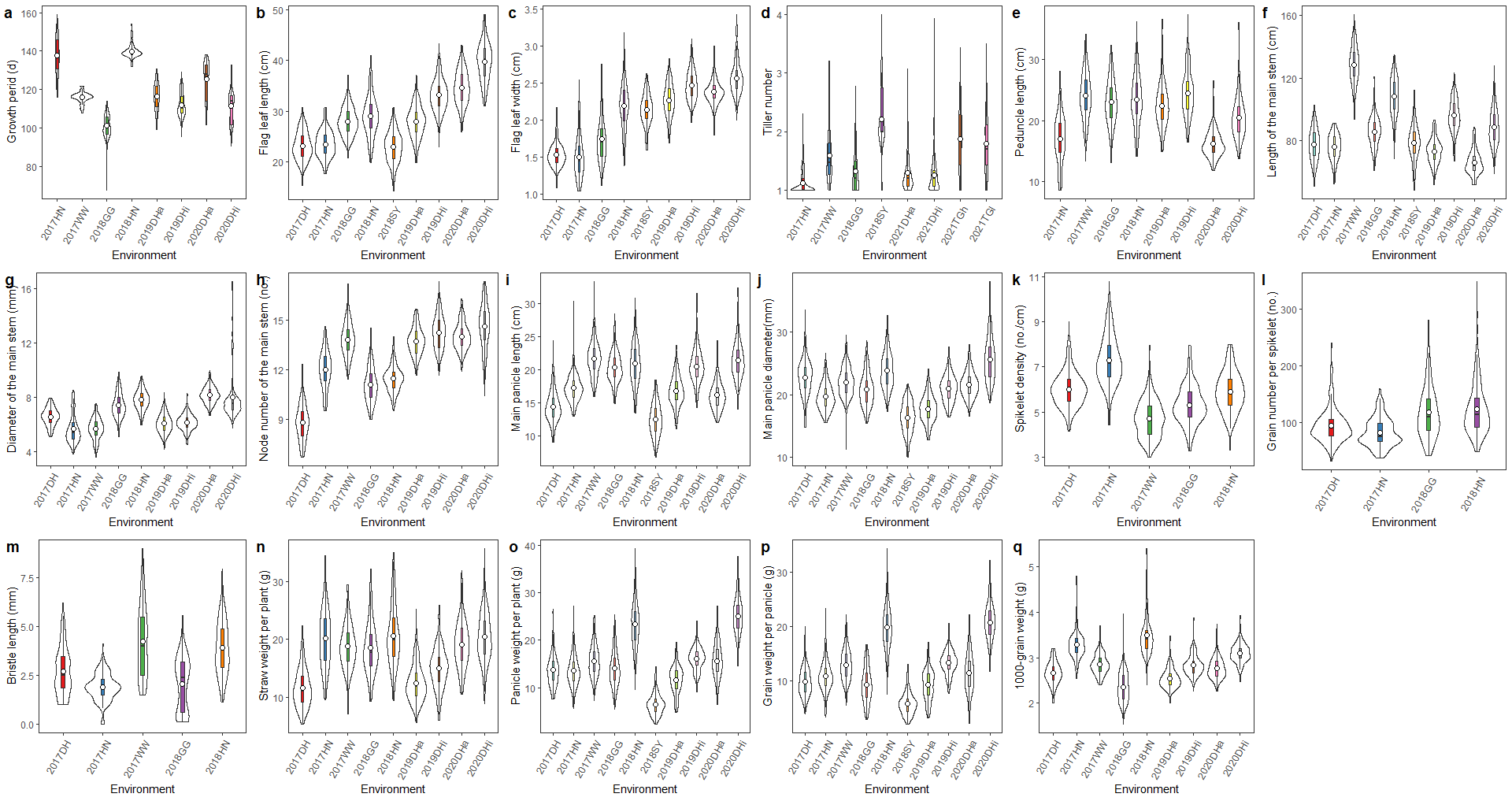

Supplement: Supplementary file 3 — Supplementary file3 (PNG 75 KB) [file 438_2022_1894_MOESM3_ESM.png]

2017DH

a

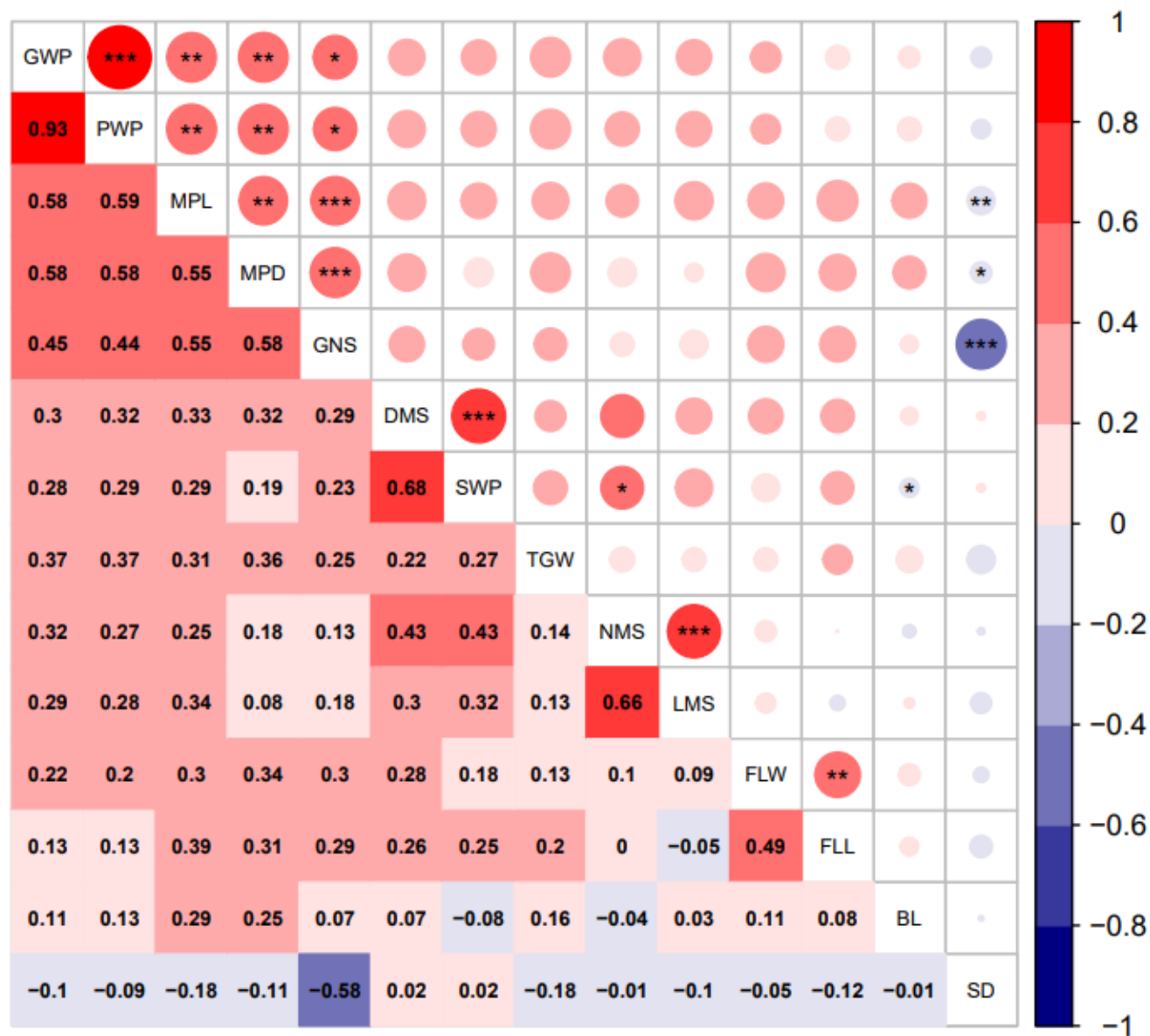

2017HN

b

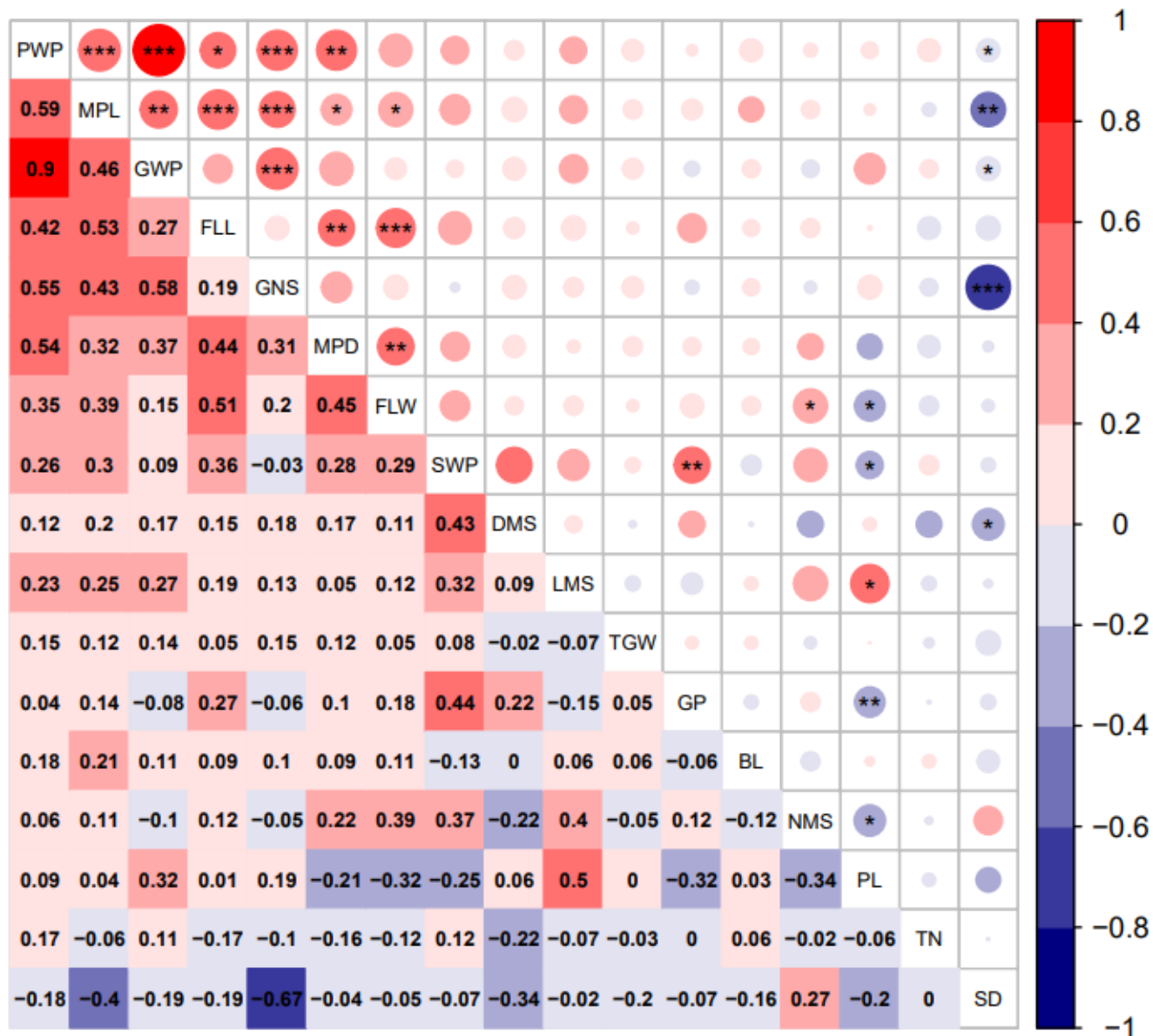

2017WW

C

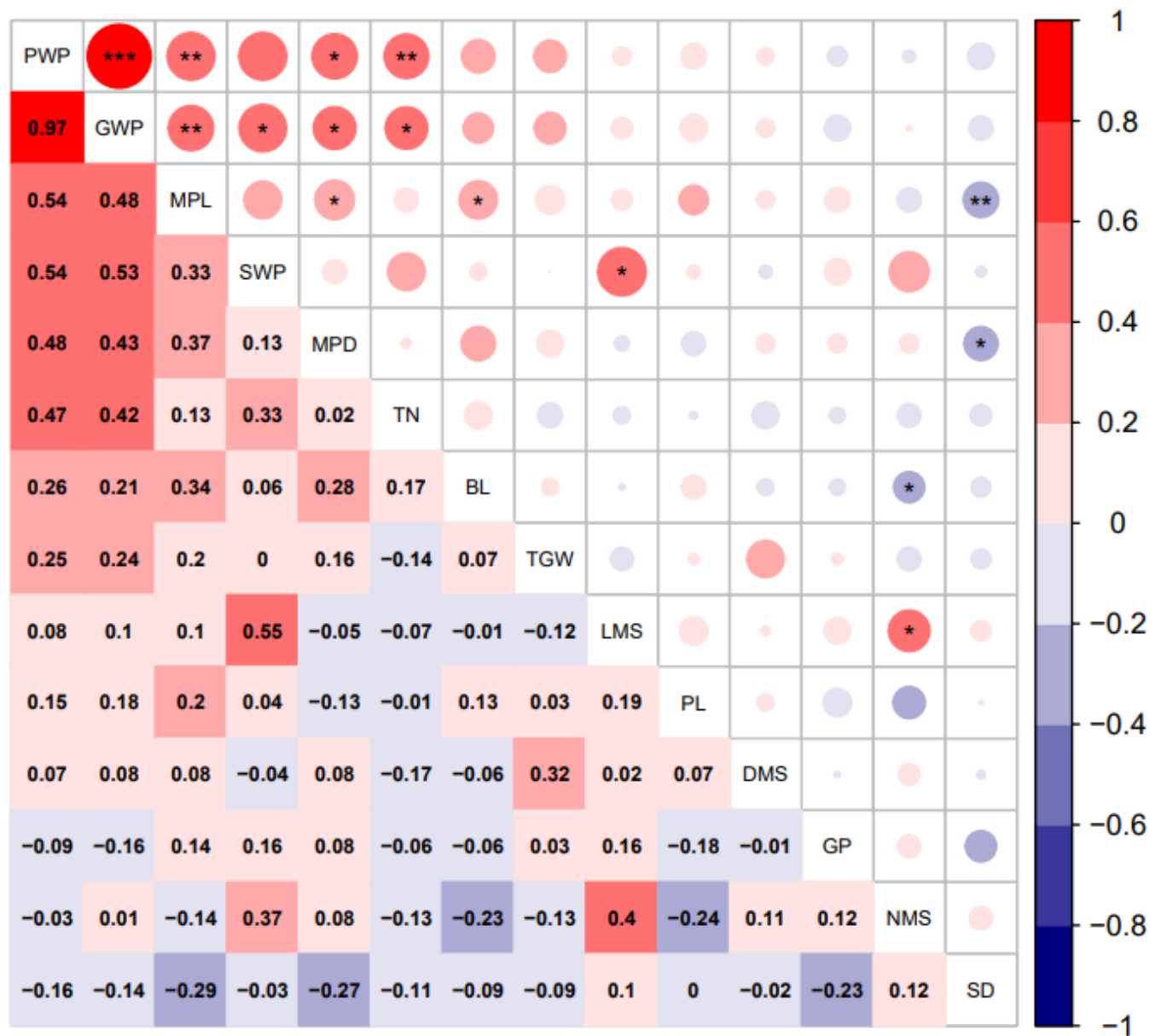

2018GG

d

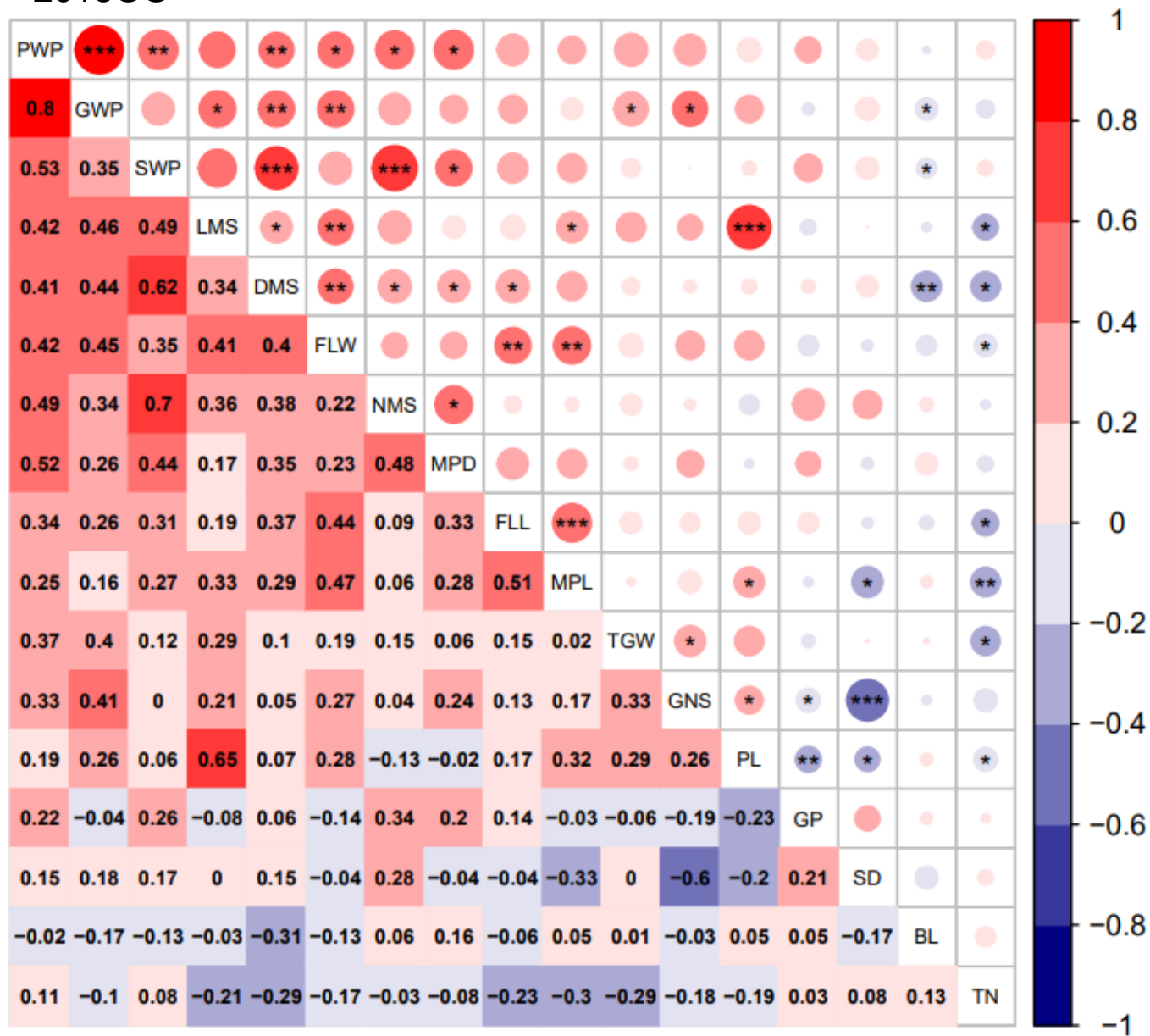

e 2018HN

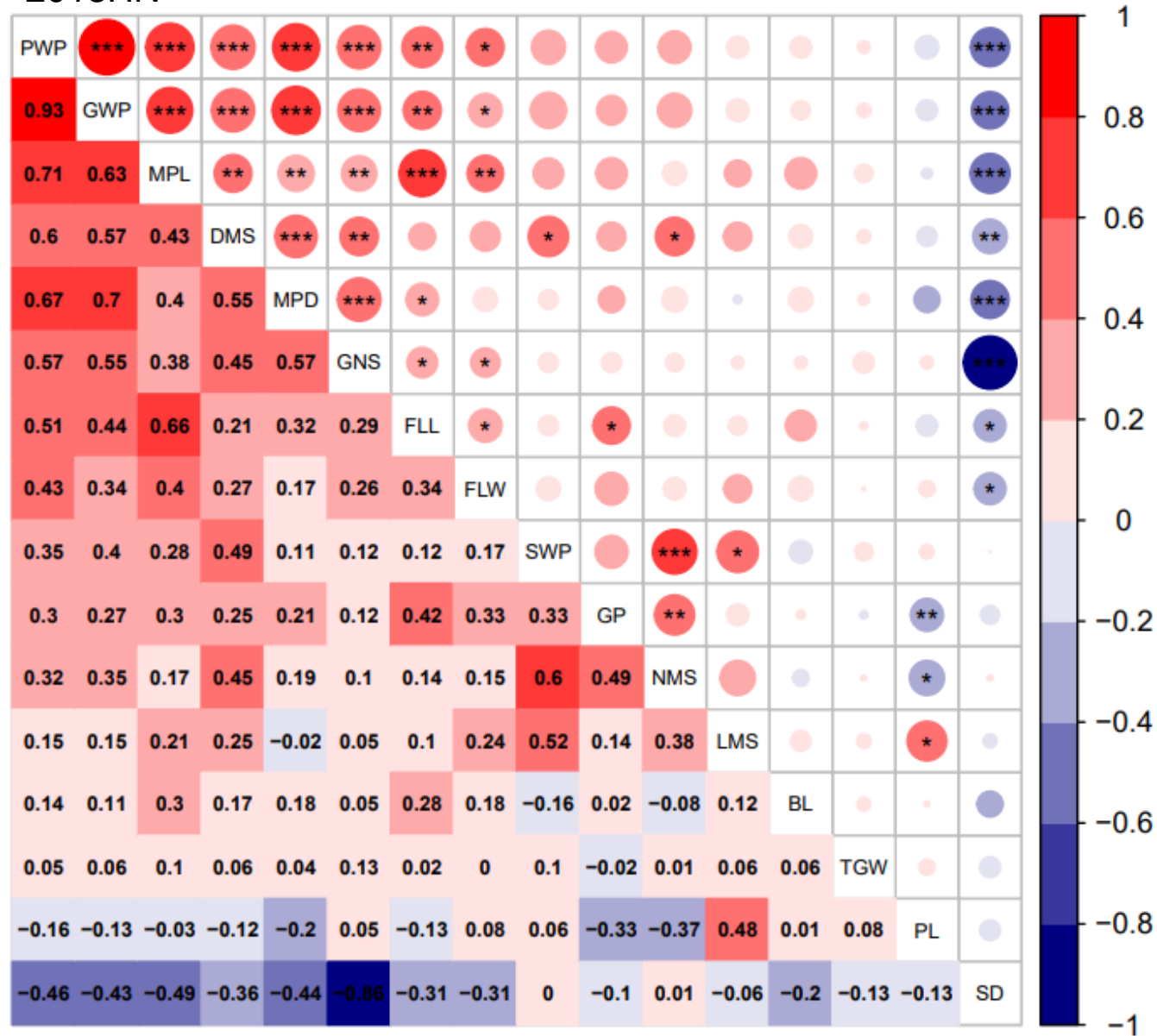

2018SY

f

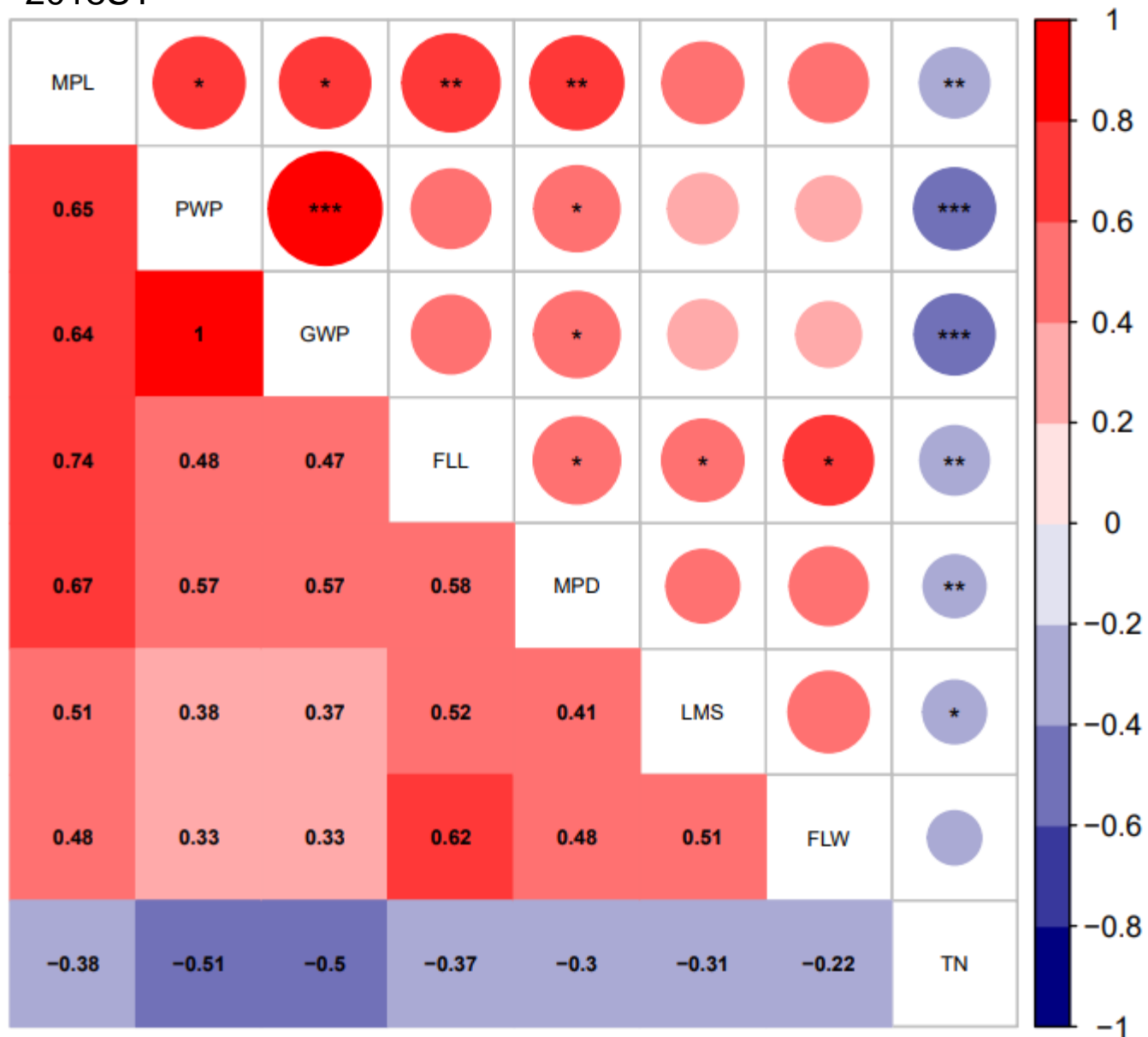

2019DHa

g

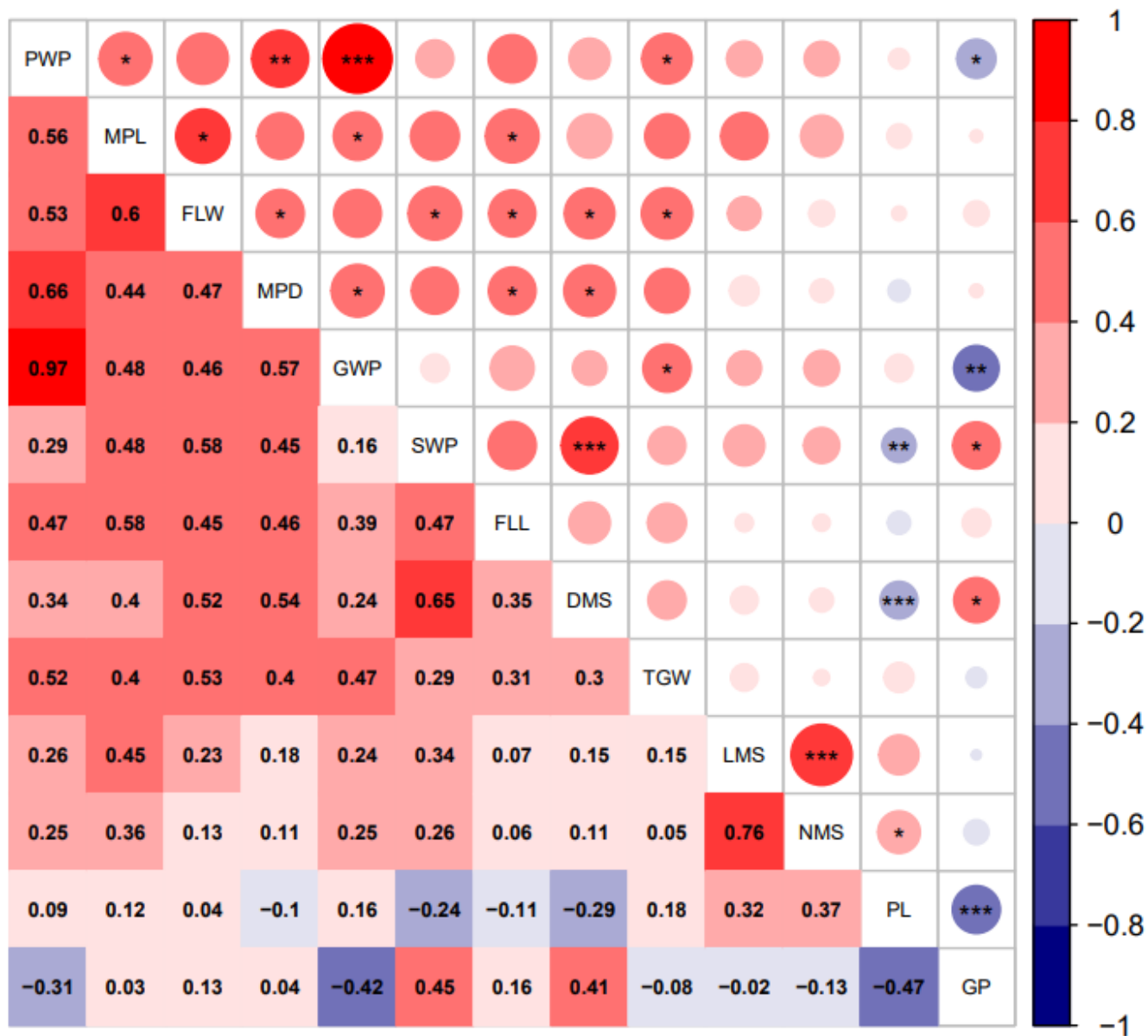

2019DHi

h

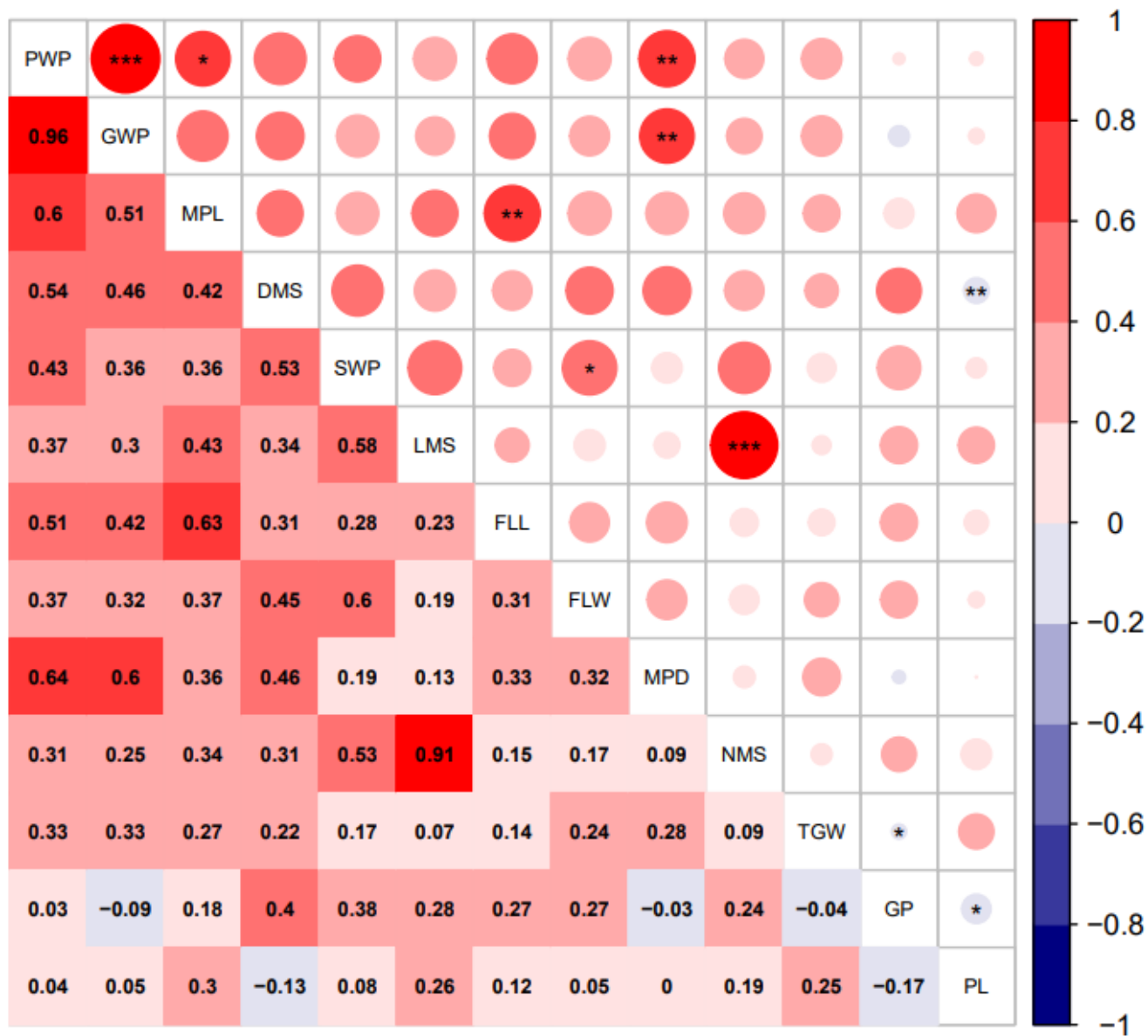

2020DHa

i

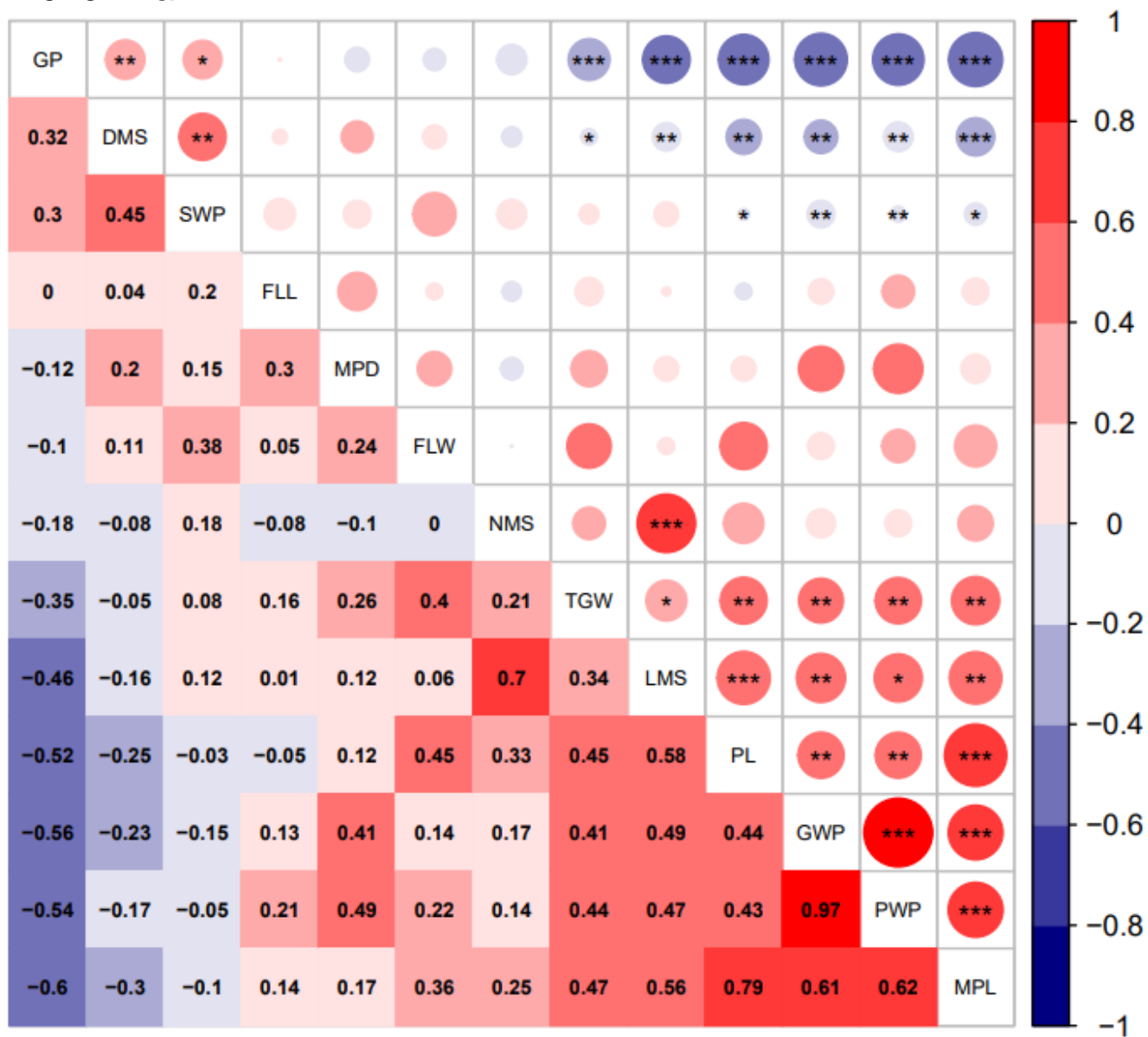

2020DHi

j

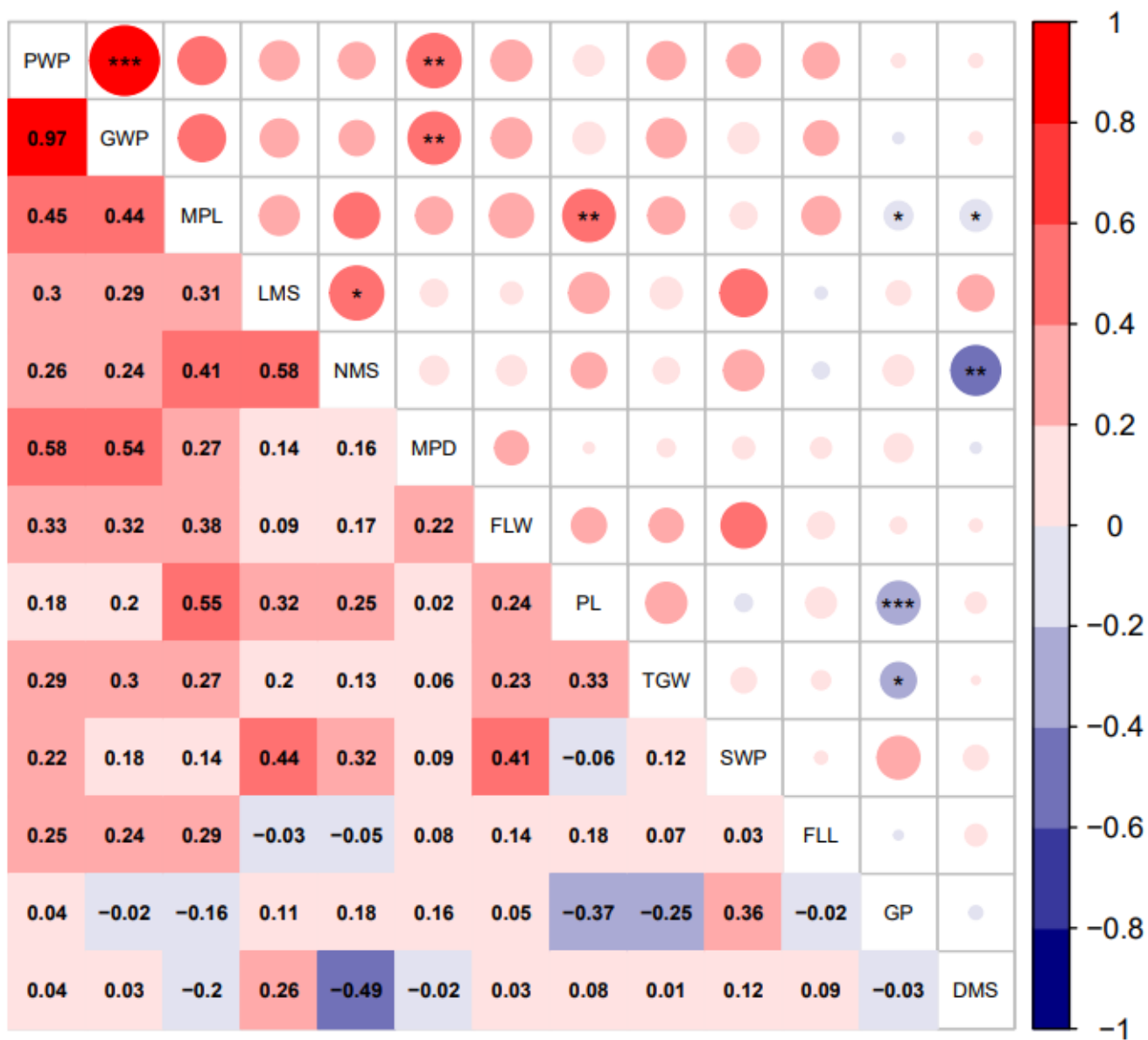

Supplement: Supplementary file 4 — Supplementary file4 (PDF 891 KB) [file 438_2022_1894_MOESM4_ESM.pdf]

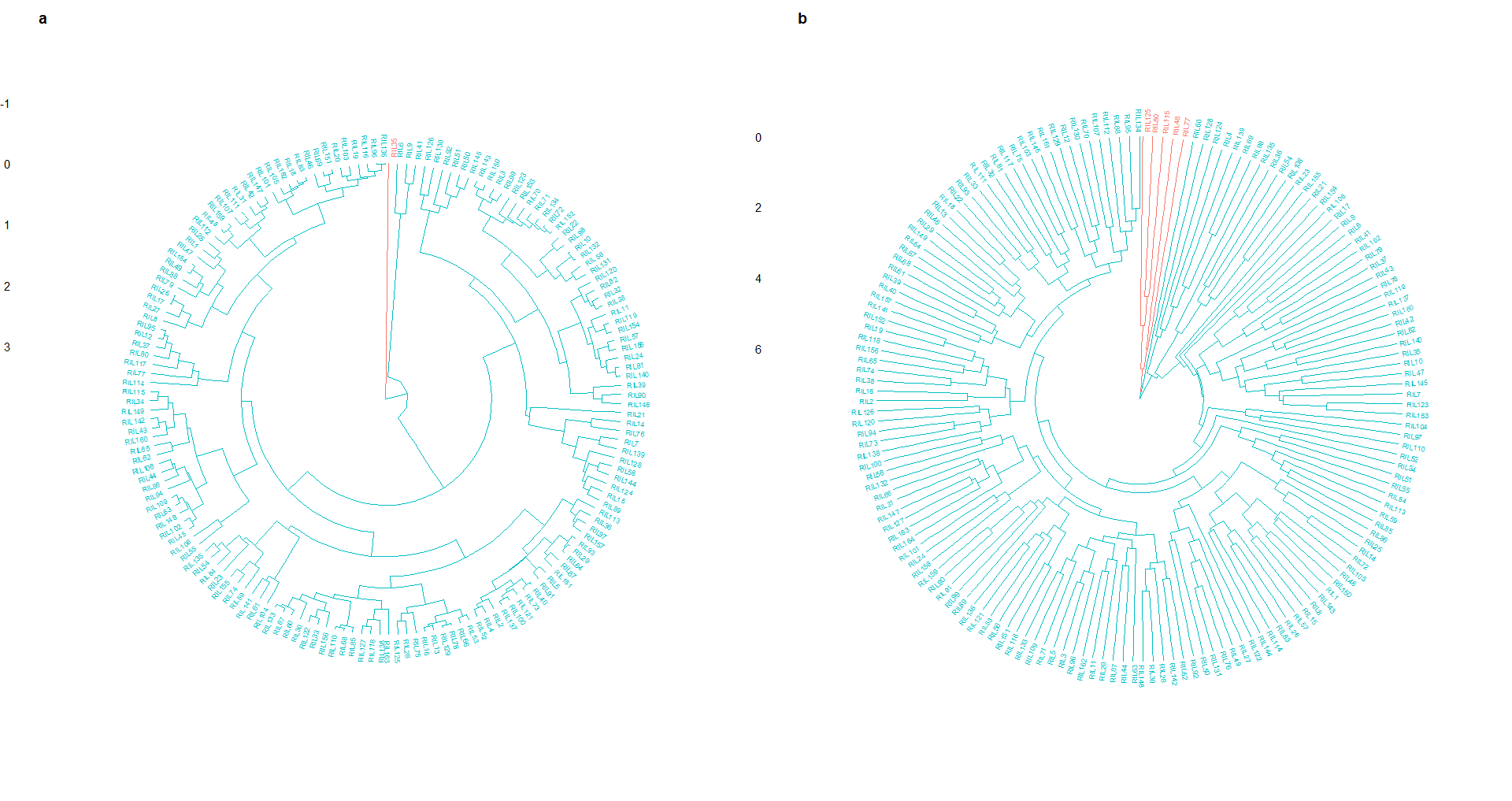

Supplement: Supplementary file 6 — Supplementary file6 (PNG 73 KB) [file 438_2022_1894_MOESM6_ESM.png]

b

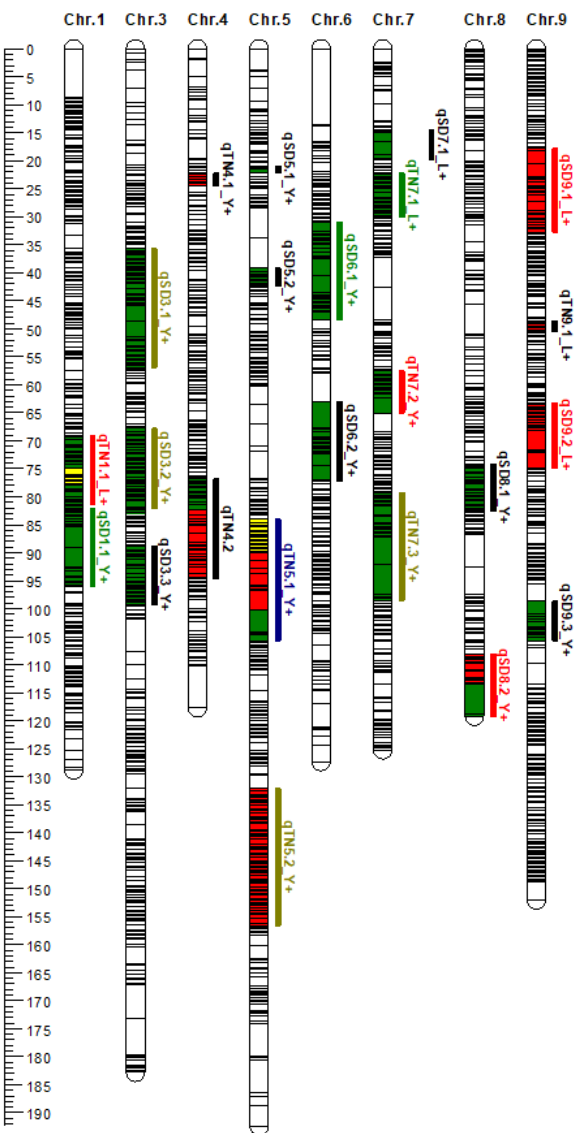

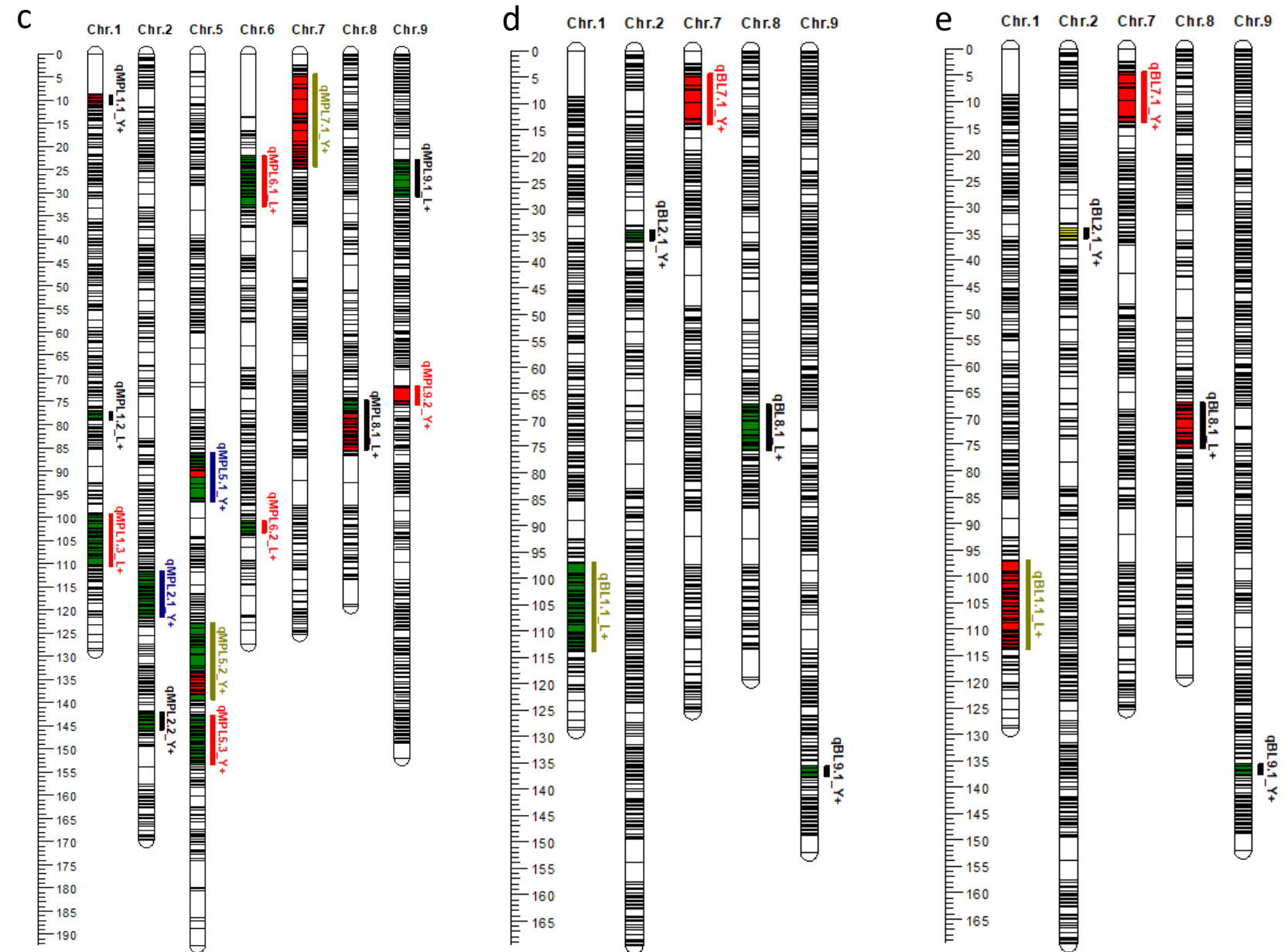

f

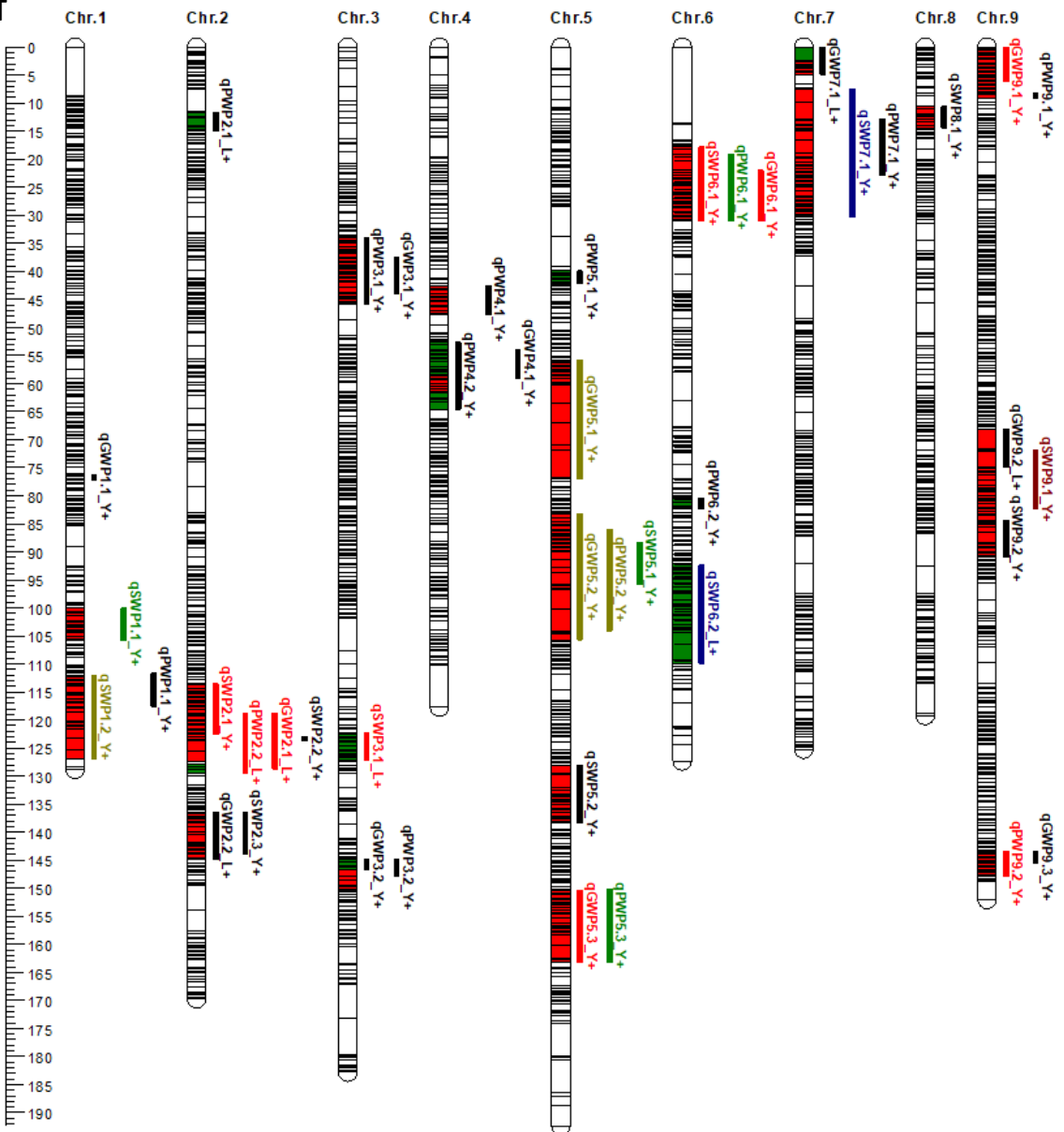

Supplement: Supplementary file 7 — Supplementary file7 (PDF 347 KB) [file 438_2022_1894_MOESM7_ESM.pdf]
