# Supplementary material for: Genome-wide identification of quantitative trait loci for morpho-agronomic and yield-related traits in foxtail millet (Setaria italica) across multi-environments
Source: Mol Genet Genomics. 2022 Apr 22;297(3):873–88. doi: 10.1007/s00438-022-01894-2 (PMC9130181; doi:10.1007/s00438-022-01894-2)
Supplement: Supplementary file 2 — Supplementary file2 (PDF 51 KB) [file 438_2022_1894_MOESM2_ESM.pdf]

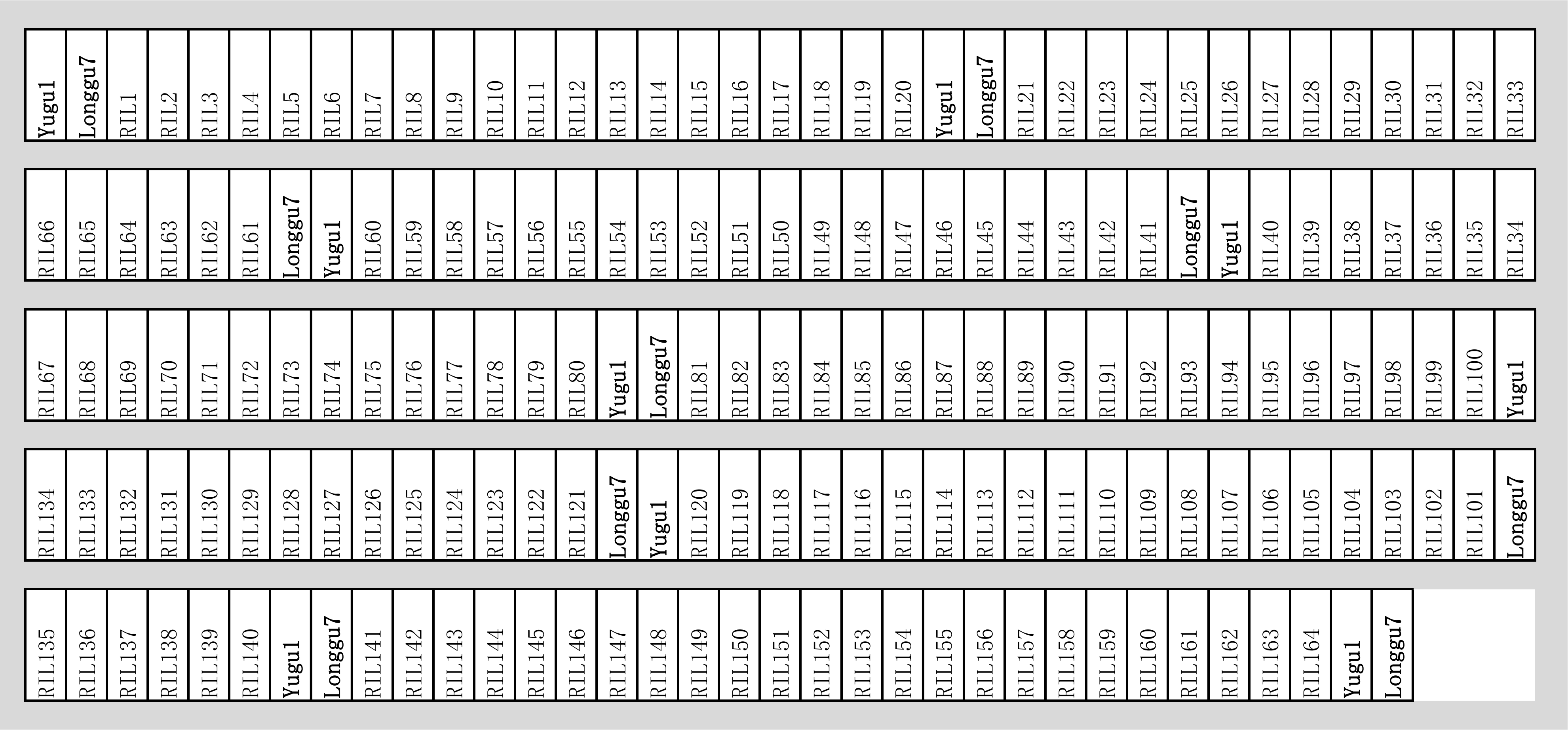

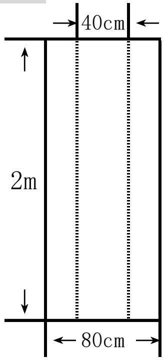

Fig. S2 Field experimental design of RIL population

Gray areas represented 70cm wide footpath in the field , and black rectangular boxes represented plots. The schematic diagram in the lower right corner showed a specific plot in the above figure, which was 2m long and 80cm wide. Each line was planted two rows in one plot (the dotted line represents the planted plants), and the distance between the two rows was for 40cm.
