# Supplementary material for: Genome-wide identification of quantitative trait loci for morpho-agronomic and yield-related traits in foxtail millet (Setaria italica) across multi-environments
Source: Mol Genet Genomics. 2022 Apr 22;297(3):873–88. doi: 10.1007/s00438-022-01894-2 (PMC9130181; doi:10.1007/s00438-022-01894-2)
Supplement: Supplementary file 8 — Supplementary file8 (DOCX 31 KB) [file 438_2022_1894_MOESM8_ESM.docx]

**Table S1** Descriptive statistics for 17 morpho-agronomic and yield-related traits in Yugu1, Longgu7 and recombinant inbred lines

| **Trait** | **Environment** |  | **Parent** | |  | **Population** | | | | |
| --- | --- | --- | --- | --- | --- | --- | --- | --- | --- | --- |
|  |  |  | **Yugu1** | **Longu7** |  | **Means** | **Range** | **Variance** | **Skewness** | **Kurtosis** |
| GP | 2017HN |  | 150.2±2.2 | 114.8±1.3 |  | 137.7±9.3 | 116.0-159.0 | 87.01 | -0.19 | -0.67 |
|  | 2017WW |  | 121.8±0.4 | 105.2±1.0 |  | 116.0±3.0 | 108.0-122.0 | 8.39 | -0.45 | 0.61 |
|  | 2018GG |  | 108.1±3.0 | 84.0±1.1 |  | 101.5±6.4 | 68.0-114.0 | 40.77 | -1.16 | 6.41 |
|  | 2018HN |  | 141.1±0.9 | 130.0±1.6 |  | 140.0±3.7 | 132.0-154.0 | 13.83 | 1.39 | 3.14 |
|  | 2019DHa |  | 117.9±0.8 | 99.4±1.2 |  | 116.6±6.6 | 99.3-130.7 | 43.15 | -0.18 | -0.39 |
|  | 2019DHi |  | 122.2±2.2 | 98.1±0.2 |  | 112.1±6.1 | 95.7-129.0 | 37.07 | 0.33 | -0.25 |
|  | 2020DHa |  | 131.9±2.1 | 97.1±0.4 |  | 125.6±10.2 | 102.0-138.0 | 103.46 | -0.91 | -0.33 |
|  | 2020DHi |  | 120.8±2.8 | 90.8±0.7 |  | 111.7±9.0 | 91.0-133.0 | 81.64 | 0.14 | -0.29 |
| FLL | 2017DH |  | 26.6±2.7 | 21.1±3.2 |  | 23.2±2.8 | 15.4-30.7 | 7.96 | 0.16 | -0.11 |
|  | 2017HN |  | 27.4±2.2 | 23.4±2.4 |  | 23.5±2.6 | 17.7-30.7 | 6.65 | 0.3 | 0.04 |
|  | 2018GG |  | 29.6±1.7 | 26.6±1.6 |  | 28.0±2.7 | 20.8-37.1 | 7.17 | 0.31 | 0.06 |
|  | 2018HN |  | 35.8±1.7 | 25.6±1.9 |  | 29.0±4.0 | 19.9-41.0 | 15.79 | 0.28 | 0.21 |
|  | 2018SY |  | 24.3±1.7 | 16.7±1.2 |  | 23.0±3.0 | 14.3-30.7 | 9.21 | -0.04 | -0.06 |
|  | 2019DHa |  | 32.83±2.73 | 27.65±4.29 |  | 28.0±3.1 | 19.9-37.1 | 9.85 | 0.02 | 0.01 |
|  | 2019DHi |  | 37.8±2.8 | 28.9±5.6 |  | 33.2±3.2 | 22.9-43.3 | 10.26 | 0.18 | 0.46 |
|  | 2020DHa |  | 39.5±3.7 | 33.3±1.7 |  | 34.7±3.3 | 25.9-43.0 | 11.11 | 0 | -0.37 |
|  | 2020DHi |  | 46.2±3.0 | 34.7±2.1 |  | 39.8±3.8 | 31.1-49.1 | 14.4 | -0.04 | -0.27 |
| FLW | 2017DH |  | 1.6±0.2 | 1.5±0.1 |  | 1.5±0.2 | 1.1-2.2 | 0.02 | 0.8 | 2.38 |
|  | 2017HN |  | 1.9±0.4 | 1.6±0.4 |  | 1.5±0.3 | 1.1-2.6 | 0.08 | 0.97 | 1.18 |
|  | 2018GG |  | 1.8±0.2 | 1.8±0.4 |  | 1.8±0.3 | 1.1-2.8 | 0.11 | 0.93 | 1 |
|  | 2018HN |  | 2.4±0.3 | 2.2±0.2 |  | 2.2±0.3 | 1.4-3.2 | 0.1 | 0.08 | 0.44 |
|  | 2018SY |  | 1.9±0.2 | 1.6±0.1 |  | 2.1±0.2 | 1.6-2.6 | 0.05 | -0.25 | 0.04 |
|  | 2019DHa |  | 2.5±0.2 | 2.4±0.2 |  | 2.3±0.2 | 1.7-2.8 | 0.05 | -0.13 | -0.25 |
|  | 2019DHi |  | 2.7±0.1 | 2.4±0.2 |  | 2.5±0.2 | 2.0-3.1 | 0.04 | 0.36 | 0.04 |
|  | 2020DHa |  | 2.6±0.2 | 2.6±0.1 |  | 2.6±0.2 | 1.8-3.0 | 0.03 | 0.71 | 2.58 |
|  | 2020DHi |  | 2.9±0.2 | 2.6±0.2 |  | 2.6±0.2 | 2.0-3.4 | 0.06 | 0.84 | 1.31 |
| TN | 2017HN |  | 1.0±0.0 | 1.0±0.3 |  | 1.1±0.2 | 1.0-2.3 | 0.05 | 2.21 | 6.16 |
|  | WW-2018 |  | 1.3±0.2 | 1.1±0.1 |  | 1.6±0.4 | 1.0-3.2 | 0.17 | 1.26 | 2.04 |
|  | 2018GG |  | 1.2±0.2 | 1.2±0.2 |  | 1.3±0.4 | 1.0-2.8 | 0.13 | 1.37 | 1.76 |
|  | 2018SY |  | 1.0±0.0 | 1.5±0.2 |  | 2.2±0.7 | 1.0-4.0 | 0.44 | 0.7 | 0.96 |
|  | 2021DHa |  | 1.2±0.2 | 1.1±0.1 |  | 1.3±0.3 | 1.0-3.1 | 0.11 | 2.6 | 9.27 |
|  | 2021DHi |  | 1.1±0.2 | 1.0±0.2 |  | 1.3±0.3 | 1.0-3.9 | 0.11 | 3.88 | 5.28 |
|  | 2021TGh |  | 1.5±0.5 | 1.5±0.5 |  | 1.8±0.5 | 1.0-3.5 | 0.27 | 0.73 | 0.55 |
|  | 2021TGl |  | 1.3±0.7 | 1.5±0.5 |  | 1.8±0.5 | 1.0-3.4 | 0.29 | 0.25 | -0.54 |
| PL | 2017HN |  | 19.6±2.1 | 24.5±3.4 |  | 17.0±4.0 | 8.6-28.1 | 15.77 | -0.06 | -0.11 |
|  | 2017WW |  | 23.3±3.0 | 31.3±2.7 |  | 24.1±3.9 | 13.3-34.2 | 14.99 | 0.1 | -0.1 |
|  | 2018GG |  | 20.8±2.5 | 28.2±2.4 |  | 23.0±3.7 | 13.1-32.3 | 13.51 | 0.06 | -0.12 |
|  | 2018HN |  | 29.8±1.2 | 32.4±1.6 |  | 23.5±4.2 | 14.1-36.2 | 17.2 | 0.22 | -0.02 |
|  | 2019DHa |  | 19.3±1.8 | 28.4±2.6 |  | 22.4±3.5 | 14.9-36.6 | 12.51 | 0.6 | 1.2 |
|  | 2019DHi |  | 26.1±1.8 | 28.4±1.5 |  | 24.5±3.8 | 16.4-37.4 | 14.77 | 0.36 | 0.17 |
|  | 2020DHa |  | 16.4±1.3 | 19.4±1.9 |  | 16.2±2.3 | 11.8-26.6 | 5.47 | 1.3 | 3.72 |
|  | 2020DHi |  | 20.1±3.2 | 23.8±2.3 |  | 20.5±3.5 | 13.8-36.1 | 12.22 | 1.22 | 3.39 |
| LMS | 2017DH |  | 83.0±9.0 | 79.5±7.3 |  | 77.5±10.1 | 50.8-102.5 | 102.55 | 0 | -0.52 |
|  | 2017HN |  | 83.1±6.6 | 81.2±8.4 |  | 75.9±8.2 | 52.4-91.3 | 66.97 | -0.4 | -0.15 |
|  | 2017WW |  | 136.1±12.7 | 121.9±5.7 |  | 128.1±12.3 | 93.7-160.6 | 150.56 | -0.26 | 0.1 |
|  | 2018GG |  | 91.1±5.9 | 83.2±4.5 |  | 85.7±9.7 | 61.1-120.8 | 94.05 | 0.06 | 0.86 |
|  | 2018HN |  | 122.1±3.6 | 100.4±5.6 |  | 108.1±12.2 | 68.7-134.8 | 148.95 | -0.37 | -0.05 |
|  | 2018SY |  | 71.8±4.8 | 57.5±7.0 |  | 78.6±11.3 | 48.5-112.3 | 128.64 | -0.08 | 0.33 |
|  | 2019DHa |  | 74.1±5.8 | 63.8±2.7 |  | 73.3±8.0 | 52.1-92.9 | 64.06 | 0.11 | -0.02 |
|  | 2019DHi |  | 99.6±4.7 | 75.3±3.3 |  | 96.0±10.4 | 67.3-123.3 | 109.01 | -0.1 | -0.05 |
|  | 2020DHa |  | 69.9±5.0 | 66.5±2.6 |  | 66.2±8.1 | 51.7-100.9 | 65.93 | 1.03 | 1.87 |
|  | 2020DHi |  | 88.5±6.2 | 71.6±6.1 |  | 88.7±11.7 | 58.8-127.7 | 135.82 | 0.42 | 0.31 |
| DMS | 2017DH |  | 7.37±0.53 | 6.04±0.65 |  | 6.59±0.64 | 5.11-7.96 | 0.41 | -0.13 | -0.42 |
|  | 2017HN |  | 6.37±0.70 | 4.64±0.75 |  | 5.67±1.03 | 3.87-8.53 | 1.07 | 1.04 | 0.52 |
|  | 2017WW |  | 6.23±1.88 | 5.16±0.43 |  | 5.70±0.78 | 3.63-7.55 | 0.6 | -0.19 | -0.02 |
|  | 2018GG |  | 8.31±0.94 | 6.52±0.47 |  | 7.41±0.90 | 5.10-9.88 | 0.81 | 0.23 | -0.05 |
|  | 2018HN |  | 8.81±0.52 | 6.71±0.66 |  | 7.81±0.71 | 6.00-9.57 | 0.51 | -0.07 | -0.13 |
|  | 2019DHa |  | 6.80±0.74 | 5.00±0.28 |  | 6.09±0.69 | 4.20-8.33 | 0.48 | 0.3 | 0.2 |
|  | 2019DHi |  | 6.83±0.29 | 5.02±0.50 |  | 6.19±0.58 | 4.57-8.27 | 0.34 | 0.31 | 1.16 |
|  | 2020DHa |  | 8.61±0.57 | 7.64±0.30 |  | 8.17±0.59 | 6.67-9.97 | 0.35 | 0.14 | 0.07 |
|  | 2020DHi |  | 8.52±1.76 | 7.45±1.92 |  | 8.01±1.93 | 5.73-16.5 | 3.74 | 2.8 | 7.83 |
| NMS | 2017DH |  | 9.6±0.8 | 8.3±1.0 |  | 8.8±1.1 | 6.7-12.3 | 1.14 | 0.29 | 0.21 |
|  | 2017HN |  | 12.2±0.9 | 10.6±0.9 |  | 12.0±1.1 | 9.5-14.6 | 1.27 | -0.14 | -0.36 |
|  | 2017WW |  | 14.9±0.8 | 10.9±1.8 |  | 13.8±1.0 | 11.4-17.2 | 1.05 | 0.21 | 0.11 |
|  | 2018GG |  | 12.3±0.7 | 9.3±0.8 |  | 11.1±1.1 | 9.0-14.5 | 1.11 | 0.48 | 0.13 |
|  | 2018HN |  | 11.4±0.2 | 10.0±0.5 |  | 11.4±0.8 | 9.5-14.0 | 0.61 | 0.4 | 0.67 |
|  | 2019DHa |  | 13.9±0.6 | 12.8±0.3 |  | 13.7±0.8 | 11.7-15.7 | 0.66 | 0.13 | -0.24 |
|  | 2019DHi |  | 14.6±0.7 | 12.4±0.6 |  | 14.3±1.0 | 11.7-17.3 | 1.01 | 0.16 | -0.2 |
|  | 2020DHa |  | 14.2±0.6 | 13.9±0.5 |  | 14.0±0.8 | 11.9-16.3 | 0.66 | 0.39 | 0.12 |
|  | 2020DHi |  | 14.8±1.1 | 13.2±1.0 |  | 14.6±1.4 | 10.4-17.3 | 1.86 | -0.34 | -0.13 |
| MPL | 2017DH |  | 14.6±2.1 | 14.8±2.6 |  | 14.4±2.4 | 9.1-24.4 | 5.85 | 0.71 | 1.46 |
|  | 2017HN |  | 21.6±1.2 | 19.8±1.3 |  | 17.3±2.1 | 13.0-30.4 | 4.53 | 1.71 | 8.65 |
|  | 2017WW |  | 19.3±2.1 | 20.1±2.2 |  | 21.7±2.6 | 16.0-33.4 | 6.79 | 0.58 | 1.76 |
|  | 2018GG |  | 21.3±1.7 | 21.4±1.1 |  | 20.5±2.3 | 14.9-28.5 | 5.39 | 0.52 | 0.82 |
|  | 2018HN |  | 27.6±0.8 | 18.8±0.9 |  | 21.0±3.2 | 13.5-31.0 | 10.42 | 0.25 | 0.24 |
|  | 2018SY |  | 14.8±1.3 | 8.4±0.7 |  | 12.5±2.5 | 6.8-18.5 | 6.01 | 0.2 | -0.35 |
|  | 2019DHa |  | 18.6±1.7 | 15.7±1.7 |  | 16.8±2.2 | 11.1-23.7 | 4.98 | 0.14 | 0.08 |
|  | 2019DHi |  | 23.3±1.7 | 16.9±1.0 |  | 20.6±2.6 | 14.3-31.6 | 6.87 | 0.63 | 1.97 |
|  | 2020DHa |  | 15.4±1.9 | 19.5±1.7 |  | 16.3±2.3 | 12.0-24.4 | 5.44 | 1.03 | 1.68 |
|  | 2020DHi |  | 22.9±2.3 | 21.8±1.7 |  | 21.5±2.70 | 14.10-32.40 | 7.16 | 0.65 | 1.38 |
| MPD | 2017DH |  | 24.82±0.26 | 20.91±0.45 |  | 22.74±2.98 | 14.81-33.53 | 8.88 | 0.06 | 0.99 |
|  | 2017HN |  | 21.45±0.90 | 16.72±0.94 |  | 19.66±2.11 | 15.52-26.68 | 4.47 | 0.62 | 0.36 |
|  | 2017WW |  | 20.59±1.31 | 18.90±0.99 |  | 21.94±2.48 | 11.29-29.47 | 6.16 | -0.26 | 1.93 |
|  | 2018GG |  | 24.61±1.63 | 17.78±0.71 |  | 20.78±2.63 | 15.38-28.59 | 6.94 | 0.45 | -0.09 |
|  | 2018HN |  | 28.69±1.71 | 20.33±0.66 |  | 23.83±2.87 | 17.26-32.67 | 8.25 | 0.45 | 0.08 |
|  | 2018SY |  | 15.13±1.82 | 12.08±0.64 |  | 16.31±2.43 | 10.00-21.67 | 5.91 | -0.22 | -0.46 |
|  | 2019DHa |  | 19.62±1.61 | 14.88±1.47 |  | 17.67±2.16 | 12.7-24.13 | 4.65 | 0.17 | 0 |
|  | 2019DHi |  | 23.58±0.82 | 16.69±0.54 |  | 20.96±2.18 | 16.43-27.63 | 4.74 | 0.27 | 0.01 |
|  | 2020DHa |  | 23.04±1.06 | 19.26±0.70 |  | 21.65±2.14 | 16.53-28.00 | 4.57 | 0.51 | 0.35 |
|  | 2020DHi |  | 28.14±2.13 | 19.77±1.54 |  | 25.63±3.47 | 18.70-38.10 | 12.04 | 0.67 | 0.57 |
| SD | 2017DH |  | 6.2±0.8 | 5.1±1.0 |  | 6.0±0.8 | 4.2-9.0 | 0.62 | 0.64 | 1.14 |
|  | 2017HN |  | 5.9±0.3 | 5.5±0.4 |  | 7.3±1.1 | 4.4-10.8 | 1.19 | 0.37 | 0.23 |
|  | 2017WW |  | 5.5±1.0 | 3.7±0.4 |  | 4.7±0.8 | 3.0-8.0 | 0.68 | 0.53 | 0.66 |
|  | 2018GG |  | 6.3±0.8 | 4.4±0.5 |  | 5.3±0.9 | 3.3-7.9 | 0.76 | 0.48 | 0.51 |
|  | 2018HN |  | 5.0±0.4 | 3.9±0.3 |  | 5.9±0.9 | 3.3-8.0 | 0.75 | 0.1 | -0.19 |
| GNS | 2017-DH |  | 106.8±21.3 | 94.1±15.7 |  | 94.9±30.8 | 32.7-241.3 | 949.72 | 1.52 | 3.95 |
|  | 2017-HN |  | 105.1±15.2 | 73.0±16.7 |  | 82.6±24.9 | 37.6-159.9 | 621.5 | 0.61 | -0.02 |
|  | 2018-GG |  | 151.9±24.2 | 109.2±19.2 |  | 118.1±45.7 | 42.1-280.4 | 2085.3 | 0.85 | 0.72 |
|  | 2018-HN |  | 146.4±42.0 | 119.3±18.6 |  | 123.1±44.5 | 49.0-294.0 | 1979.35 | 1.05 | 1.23 |
| BL | 2017-DH |  | 2.08±0.71 | 2.75±1.17 |  | 2.71±1.20 | 1.00-6.20 | 1.44 | 0.54 | -0.35 |
|  | 2017-HN |  | 1.77±0.69 | 2.23±0.89 |  | 1.89±0.69 | 0.10-4.13 | 0.47 | -0.09 | 0.8 |
|  | 2017-WW |  | 3.61±1.17 | 4.44±0.83 |  | 4.25±1.81 | 1.50-9.00 | 3.27 | 0.35 | -0.71 |
|  | 2018-GG |  | 2.79±2.04 | 3.02±1.71 |  | 2.06±1.37 | 0.10-5.57 | 1.89 | 0.02 | -1.07 |
|  | 2018-HN |  | 3.57±0.68 | 3.94±0.55 |  | 3.90±1.37 | 1.13-7.96 | 1.88 | 0.28 | -0.36 |
| SWP | 2017DH |  | 15.54±2.53 | 9.08±4.73 |  | 11.59±3.27 | 5.41-22.31 | 10.71 | 0.63 | 0.22 |
|  | 2017HN |  | 23.82±2.87 | 13.77±2.88 |  | 20.14±4.88 | 9.66-34.48 | 23.84 | 0.19 | -0.19 |
|  | 2017WW |  | 17.61±3.26 | 9.37±3.02 |  | 18.83±3.85 | 7.20-29.40 | 14.82 | 0.33 | 0.57 |
|  | 2018GG |  | 25.45±3.84 | 12.35±2.07 |  | 18.57±4.56 | 9.27-32.10 | 20.76 | 0.65 | 0.5 |
|  | 2018HN |  | 27.15±3.99 | 16.87±3.20 |  | 20.66±5.23 | 9.42-35.05 | 27.38 | 0.46 | -0.1 |
|  | 2019DHa |  | 19.49±2.94 | 10.17±1.72 |  | 12.52±3.27 | 5.73-25.17 | 10.71 | 0.91 | 1.17 |
|  | 2019DHi |  | 19.53±1.51 | 12.12±2.23 |  | 14.99±3.48 | 6.07-25.97 | 12.09 | 0.16 | 0.46 |
|  | 2020DHa |  | 26.91±4.43 | 15.49±1.84 |  | 19.17±4.07 | 10.53-31.87 | 16.56 | 0.37 | 0.32 |
|  | 2020DHi |  | 25.99±2.27 | 13.99±1.81 |  | 20.5±4.32 | 8.90-35.70 | 18.66 | 0.34 | 0.59 |
| PWP | 2017DH |  | 16.91±3.57 | 12.35±5.47 |  | 13.74±3.44 | 7.57-26.58 | 11.84 | 0.95 | 1.85 |
|  | 2017HN |  | 21.19±3.27 | 11.83±2.41 |  | 13.52±3.34 | 5.56-27.16 | 11.13 | 0.68 | 1.6 |
|  | 2017WW |  | 11.81±2.94 | 10.64±3.60 |  | 15.65±3.34 | 7.33-25.20 | 11.19 | 0.42 | 0.13 |
|  | 2018GG |  | 19.21±3.22 | 12.21±1.89 |  | 14.02±3.71 | 5.38-25.37 | 13.79 | 0.5 | 0.1 |
|  | 2018HN |  | 34.37±3.13 | 16.94±1.61 |  | 23.40±4.90 | 9.65-39.34 | 24.04 | 0.35 | 0.54 |
|  | 2018SY |  | 13.09±2.21 | 8.57±1.57 |  | 6.48±2.27 | 2.30-14.44 | 5.16 | 0.9 | 1.26 |
|  | 2019DHa |  | 12.89±2.82 | 11.00±1.96 |  | 11.63±2.95 | 4.83-19.57 | 8.7 | -0.13 | -0.1 |
|  | 2019DHi |  | 17.54±1.27 | 11.70±1.63 |  | 16.04±2.51 | 9.03-24.00 | 6.3 | -0.12 | 0.62 |
|  | 2020DHa |  | 18.13±3.24 | 17.44±0.79 |  | 15.68±3.72 | 6.40-27.27 | 13.82 | 0.14 | 0.21 |
|  | 2020DHi |  | 30.32±2.26 | 18.92±1.34 |  | 25.02±4.01 | 14.63-37.70 | 16.05 | 0.03 | 0.51 |
| GWP | 2017DH |  | 13.25±3.01 | 8.86±3.75 |  | 9.93±2.67 | 4.01-19.94 | 7.13 | 0.79 | 1.22 |
|  | 2017HN |  | 16.71±2.24 | 9.97±2.18 |  | 10.82±2.83 | 3.38-23.36 | 7.99 | 0.73 | 2.55 |
|  | 2017WW |  | 9.36±2.67 | 8.17±2.26 |  | 12.94±2.91 | 5.53-22.20 | 8.47 | 0.34 | 0.26 |
|  | 2018GG |  | 12.52±2.49 | 10.15±1.92 |  | 9.27±3.15 | 2.91-16.65 | 9.9 | 0.2 | -0.64 |
|  | 2018HN |  | 31.25±4.48 | 14.13±1.39 |  | 19.82±4.03 | 7.50-34.29 | 16.27 | 0.29 | 0.91 |
|  | 2018SY |  | 11.01±2.21 | 8.57±1.57 |  | 5.8±2.06 | 2.09-12.98 | 4.26 | 0.86 | 1.15 |
|  | 2019DHa |  | 9.34±2.34 | 9.46±1.80 |  | 9.28±2.80 | 3.23-17.03 | 7.84 | 0.05 | -0.26 |
|  | 2019DHi |  | 13.32±1.30 | 9.96±1.31 |  | 13.34±2.20 | 7.67-20.50 | 4.83 | -0.02 | 0.57 |
|  | 2020DHa |  | 13.21±2.54 | 14.15±0.79 |  | 11.45±3.60 | 2.20-22.20 | 12.96 | 0.18 | 0.07 |
|  | 2020DHi |  | 24.35±1.97 | 16.11±1.23 |  | 20.74±3.53 | 11.77-32.17 | 12.46 | 0.05 | 0.59 |
| TGW | 2017DH |  | 2.92±0.10 | 2.64±0.12 |  | 2.66±0.24 | 2.00-3.20 | 0.06 | -0.06 | 0.01 |
|  | 2017HN |  | 3.90±0.20 | 3.42±0.24 |  | 3.29±0.33 | 2.54-4.79 | 0.11 | 1.73 | 6.18 |
|  | 2017WW |  | 2.99±0.22 | 2.56±0.33 |  | 2.86±0.23 | 2.40-3.70 | 0.05 | 0.54 | 0.64 |
|  | 2018GG |  | 2.75±0.28 | 2.61±0.22 |  | 2.36±0.33 | 1.54-3.97 | 0.11 | 0.69 | 2.23 |
|  | 2018HN |  | 3.77±0.22 | 3.66±0.07 |  | 3.49±0.45 | 2.40-5.40 | 0.21 | 1.52 | 3.56 |
|  | 2019DHa |  | 2.80±0.19 | 2.71±0.09 |  | 2.54±0.22 | 2.00-3.47 | 0.05 | 1.15 | 2.98 |
|  | 2019DHi |  | 3.04±0.18 | 2.92±0.06 |  | 2.84±0.24 | 2.27-3.87 | 0.06 | 1.23 | 3.18 |
|  | 2020DHa |  | 3.09±0.24 | 2.99±0.13 |  | 2.77±0.23 | 2.27-3.73 | 0.05 | 0.92 | 1.84 |
|  | 2020DHi |  | 3.37±0.10 | 3.01±0.07 |  | 3.10±0.21 | 2.47-3.93 | 0.04 | 0.29 | 1.28 |
