# Supplementary material for: Genome-wide identification of quantitative trait loci for morpho-agronomic and yield-related traits in foxtail millet (Setaria italica) across multi-environments
Source: Mol Genet Genomics. 2022 Apr 22;297(3):873–88. doi: 10.1007/s00438-022-01894-2 (PMC9130181; doi:10.1007/s00438-022-01894-2)
Supplement: Supplementary file 9 — Supplementary file9 (DOCX 17 KB) [file 438_2022_1894_MOESM9_ESM.docx]

**Table S2** Analysis of variance (ANOVA) of 17 morpho-agronomic and yield-related traits of RIL population under multi-environment

| **Trait** | **Factor** | **Sum of squares** | **df** | **Mean square** | **F** |
| --- | --- | --- | --- | --- | --- |
| GP | Enviroment | 204394.20 | 7 | 29199.17 | 928.63^**^ |
|  | Genotype | 31673.61 | 163 | 194.32 | 6.18^**^ |
|  | Error | 35782.42 | 1138 | 31.44 |  |
| FLL | Enviroment | 43945.16 | 8 | 5493.14 | 870.51^**^ |
|  | Genotype | 6819.33 | 163 | 41.84 | 6.63^**^ |
|  | Error | 8209.62 | 1301 | 6.31 |  |
| FLW | Enviroment | 210.16 | 8 | 26.27 | 559.54^**^ |
|  | Genotype | 27.09 | 163 | 0.17 | 3.54^**^ |
|  | Error | 61.08 | 1301 | 0.05 |  |
| TN | Enviroment | 155.39 | 7 | 22.20 | 146.26^**^ |
|  | Genotype | 78.49 | 163 | 0.48 | 3.17^**^ |
|  | Error | 171.94 | 1133 | 0.15 |  |
| PL | Enviroment | 11776.33 | 7 | 1682.33 | 210.98^**^ |
|  | Genotype | 8258.12 | 163 | 50.66 | 6.35^**^ |
|  | Error | 9074.24 | 1138 | 7.97 |  |
| LMS | Enviroment | 512061.48 | 9 | 56895.72 | 954.94^**^ |
|  | Genotype | 85162.40 | 163 | 522.47 | 8.77^**^ |
|  | Error | 87166.21 | 1463 | 59.58 |  |
| DMS | Enviroment | 1325.41 | 8 | 165.68 | 203.64^**^ |
|  | Genotype | 291.73 | 163 | 1.79 | 2.20^**^ |
|  | Error | 1057.65 | 1300 | 0.81 |  |
| NMS | Enviroment | 4858.03 | 8 | 607.25 | 787.38^**^ |
|  | Genotype | 515.07 | 163 | 3.16 | 4.10^**^ |
|  | Error | 1001.83 | 1299 | 0.77 |  |
| MPL | Enviroment | 15404.26 | 9 | 1711.58 | 429.34^**^ |
|  | Genotype | 4476.20 | 163 | 27.46 | 6.89^**^ |
|  | Error | 5808.40 | 1457 | 3.99 |  |
| MPD | Enviroment | 11171.01 | 9 | 1241.22 | 331.86^**^ |
|  | Genotype | 5295.13 | 163 | 32.49 | 8.69^**^ |
|  | Error | 5449.41 | 1457 | 3.74 |  |
| SD | Enviroment | 606.65 | 4 | 151.66 | 328.71^**^ |
|  | Genotype | 350.47 | 163 | 2.15 | 4.66^**^ |
|  | Error | 297.60 | 645 | 0.46 |  |
| GNS | Enviroment | 4458.89 | 3 | 1486.30 | 199.72^**^ |
|  | Genotype | 3097.40 | 163 | 19.00 | 2.55^**^ |
|  | Error | 3586.99 | 482 | 7.44 |  |
| BL | Enviroment | 734.11 | 4 | 183.53 | 140.91^**^ |
|  | Genotype | 596.71 | 163 | 3.66 | 2.81^**^ |
|  | Error | 811.44 | 623 | 1.30 |  |
| SWP | Enviroment | 15914.41 | 8 | 1989.30 | 209.52^**^ |
|  | Genotype | 12864.51 | 163 | 78.92 | 8.31^**^ |
|  | Error | 12323.70 | 1298 | 9.49 |  |
| PWP | Enviroment | 42259.20 | 9 | 4695.47 | 469.31^**^ |
|  | Genotype | 5157.97 | 163 | 31.64 | 3.16^**^ |
|  | Error | 14557.45 | 1455 | 10.01 |  |
| GWP | Enviroment | 32370.07 | 9 | 3596.67 | 450.83 ^**^ |
|  | Genotype | 3303.98 | 163 | 20.27 | 2.54^**^ |
|  | Error | 11607.71 | 1455 | 7.98 |  |
| TGW | Enviroment | 168.54 | 8 | 21.07 | 333.02^**^ |
|  | Genotype | 37.69 | 163 | 0.23 | 3.65^**^ |
|  | Error | 81.93 | 1295 | 0.06 |  |
