# Supplementary material for: Genome-wide identification of quantitative trait loci for morpho-agronomic and yield-related traits in foxtail millet (Setaria italica) across multi-environments
Source: Mol Genet Genomics. 2022 Apr 22;297(3):873–88. doi: 10.1007/s00438-022-01894-2 (PMC9130181; doi:10.1007/s00438-022-01894-2)
Supplement: Supplementary file 10 — Supplementary file10 (DOCX 16 KB) [file 438_2022_1894_MOESM10_ESM.docx]

**Table S3** Heritability for 17 morpho-agronomic and yield-related traits detected on multi-environments

| **Trait** | **Vg** | **Vge** | **Vgy** | **Ve** | **L** | **Y** | **H** |
| --- | --- | --- | --- | --- | --- | --- | --- |
| GP | 19.523 | 0.000 | 3.468 | 31.621 | 5 | 4 | 0.889 |
| FLL | 3.6940 | 0.4353 | 1.0468 | 5.0728 | 5 | 4 | 0.860 |
| FLW | 0.011584 | 0.006335 | 0.004585 | 0.037706 | 5 | 4 | 0.729 |
| TN | 3.451e-02 | 9.754e-02 | 1.838e-02 | 4.176e-02 | 8 | 3 | 0.632 |
| PL | 5.1379 | 0.4854 | 1.0220 | 6.8258 | 5 | 4 | 0.881 |
| LMS | 45.008 | 7.762 | 2.716 | 50.415 | 6 | 4 | 0.917 |
| DMS | 1.085e-01 | 0.000e+00 | 5.208e-10 | 8.157e-01 | 5 | 4 | 0.727 |
| NMS | 0.23579 | 0.11531 | 0.06444 | 0.61712 | 5 | 4 | 0.771 |
| MPL | 2.2484 | 0.1621 | 0.4853 | 3.4999 | 6 | 4 | 0.884 |
| MPD | 2.7988 | 0.1466 | 0.2909 | 3.3839 | 6 | 4 | 0.922 |
| SD | 3.231e-01 | 8.455e-02 | 5.891e-10 | 3.866e-01 | 4 | 2 | 0.823 |
| GNS | 2.918e+00 | 0.000e+00 | 1.328e-09 | 7.433e+00 | 3 | 2 | 0.702 |
| BL | 0.46073 | 0.08223 | 0.07825 | 1.17545 | 4 | 2 | 0.690 |
| SWP | 7.5127 | 0.6405 | 1.0965 | 8.0591 | 5 | 4 | 0.903 |
| PWP | 1.9303 | 0.4615 | 0.5526 | 9.8279 | 6 | 4 | 0.756 |
| GWP | 1.0473 | 0.4841 | 0.1341 | 8.2201 | 6 | 4 | 0.696 |
| TGW | 0.016865 | 0.006589 | 0.005490 | 0.053044 | 5 | 4 | 0.759 |

H: heritability. Vg, Vge, Vgy and Ve are genetic variance, variance of interaction between genotype and environment, variance of interaction between genotype and year and environmental variance, respectively. L and Y are the number of environments and years, respectively.
