# Supplementary material for: Genome-wide identification of quantitative trait loci for morpho-agronomic and yield-related traits in foxtail millet (Setaria italica) across multi-environments
Source: Mol Genet Genomics. 2022 Apr 22;297(3):873–88. doi: 10.1007/s00438-022-01894-2 (PMC9130181; doi:10.1007/s00438-022-01894-2)
Supplement: Supplementary file 12 — Supplementary file12 (DOCX 15 KB) [file 438_2022_1894_MOESM12_ESM.docx]

**Table S5** SNP/InDels distribution and density on nine chromosomes between parental lines

| Chromosome | Length | No. SNPs | SNP density | No. InDels | InDel density |
| --- | --- | --- | --- | --- | --- |
| Chr.1 | 42,145,699 | 87,346 | 2072.48 | 12,717 | 301.74 |
| Chr.2 | 49,199,997 | 170,948 | 3474.55 | 21,913 | 445.39 |
| Chr.3 | 50,651,951 | 165,815 | 3273.62 | 21,627 | 426.97 |
| Chr.4 | 40,407,879 | 76,305 | 1888.37 | 10,703 | 264.87 |
| Chr.5 | 47,252,588 | 134,675 | 2850.11 | 18,094 | 382.92 |
| Chr.6 | 36,014,550 | 113,887 | 3162.25 | 15,952 | 442.93 |
| Chr.7 | 35,964,315 | 177,287 | 4929.53 | 21,011 | 584.22 |
| Chr.8 | 40,689,132 | 254,692 | 6259.46 | 27,610 | 678.56 |
| Chr.9 | 58,970,307 | 144,644 | 2452.83 | 18,191 | 308.48 |
| Whole | 401,296,418 | 1,325,599 | 3303.29 | 167,818 | 418.19 |
