# Supplementary material for: Genome-wide identification of quantitative trait loci for morpho-agronomic and yield-related traits in foxtail millet (Setaria italica) across multi-environments
Source: Mol Genet Genomics. 2022 Apr 22;297(3):873–88. doi: 10.1007/s00438-022-01894-2 (PMC9130181; doi:10.1007/s00438-022-01894-2)
Supplement: Supplementary file 14 — Supplementary file14 (DOCX 18 KB) [file 438_2022_1894_MOESM14_ESM.docx]

**Table S7** Phenotypic characteristics of superiority lines selected from (Yugu1×Longgu7) RIL population

| Trait | Longgu7 | Yuggu1 | RIL35 | RIL48 | RIL77 | RIL80 | RIL115 | RIL125 |
| --- | --- | --- | --- | --- | --- | --- | --- | --- |
| Growth period (d) | 102.4±14.4 | 126.7±13.6 | 126.2±12.2 | 113.1±22.4 | 123.5±12.0 | 121.3±14.1 | 126.5±15.1 | 122.8±15.8 |
| Flag leaf length (cm) | 26.3±5.8 | 33.4±7.1 | 30.1±6.4 | 29.7±6.2 | 35.2±6.8 | 31.8±6.2 | 31.7±5.9 | 31.1±5.6 |
| Flag leaf width (cm) | 2.1±0.5 | 2.2±0.5 | 2.1±0.4 | 2.4±0.6 | 2.1±0.5 | 2.6±0.6 | 2.5±0.4 | 2.2±0.4 |
| Length of the main stem (cm) | 81.1±19.0 | 90.9±22.4 | 65.0±12.8 | 91.5±20.6 | 92.8±21.0 | 93.5±19.1 | 93.2±20.7 | 85.8±18.8 |
| Diameter of the main stem (mm) | 7.55±4.39 | 9.28±4.45 | 7.40±1.29 | 7.47±1.19 | 6.62±0.70 | 6.54±1.11 | 7.73±1.25 | 7.27±0.83 |
| Peduncle length (cm) | 27.0±4.3 | 21.9±4.3 | 16.3±3.3 | 24.1±4.3 | 27.1±6.7 | 25.1±4.8 | 22.7±5.8 | 21.3±2.6 |
| Node of the main stem (no.) | 11.3±2.0 | 13.0±1.8 | 11.4±1.8 | 12.3±2.2 | 12.4±2.2 | 12.8±2.0 | 13.1±2.0 | 12.6±2.1 |
| Tiller number | 1.1±0.1 | 1.3±0.4 | 1.6±0.3 | 1.3±0.4 | 1.3±0.3 | 1.2±0.3 | 1.3±0.3 | 1.8±0.8 |
| Main panicle length (cm) | 17.67±4.0 | 20.0±4.2 | 16.9±3.4 | 21.9±4.4 | 26.0±7.3 | 22.4±5.5 | 19.6±3.5 | 22.0±3.5 |
| Main panicle diameter (mm) | 21.98±14.42 | 23.84±13.32 | 22.91±3.82 | 18.49±2.53 | 21.52±4.52 | 24.14±3.44 | 25.09±4.49 | 25.66±5.78 |
| Spikelet density (no./cm) | 4.8±0.8 | 5.5±1.0 | 6.4±1.4 | 4.9±0.5 | 4.4±0.7 | 5.1±1.3 | 5.0±1.2 | 4.2±0.7 |
| Grain number per spikelet (no.) | 102.1±26.2 | 138.5±37.8 | 84.5±63.5 | 99.2±21.1 | 97.3±38.4 | 126.9±36.5 | 164.2±96.9 | 169.3±68.8 |
| Bristle length (mm) | 3.23±0.83 | 2.83±0.94 | 2.99±1.23 | 4.56±1.18 | 2.88±0.66 | 1.89±0.73 | 2.59±1.57 | 3.67±0.83 |
| Straw weight per plant (g) | 12.50±2.83 | 22.36±4.36 | 15.03±2.84 | 21.57±6.58 | 18.10±3.55 | 19.52±5.40 | 25.98±4.29 | 18.17±3.89 |
| Panicle weight per plant (g) | 13.40±3.48 | 19.35±7.65 | 16.31±7.11 | 15.14±6.49 | 18.38±7.85 | 19.09±9.13 | 19.49±7.21 | 18.63±7.54 |
| Grain weight per the main panicle (g) | 10.88±2.86 | 15.24±7.18 | 12.70±6.33 | 13.24±5.11 | 13.35±7.45 | 15.17±8.41 | 15.85±5.96 | 13.68±6.95 |
| 1000-grain weight (g) | 2.96±0.37 | 3.15±0.45 | 2.68±0.36 | 2.83±0.49 | 2.97±0.31 | 3.04±0.35 | 3.04±0.43 | 3.21±0.97 |

The phenotypic values were presented by mean ±standard deviation.

Red denoted positive transgressive inheritance, green indicated negative transgressive inheritance.
