# Supplementary material for: Genome-wide identification of quantitative trait loci for morpho-agronomic and yield-related traits in foxtail millet (Setaria italica) across multi-environments
Source: Mol Genet Genomics. 2022 Apr 22;297(3):873–88. doi: 10.1007/s00438-022-01894-2 (PMC9130181; doi:10.1007/s00438-022-01894-2)
Supplement: Supplementary file 5 — Supplementary file5 (PDF 108 KB) [file 438_2022_1894_MOESM5_ESM.pdf]

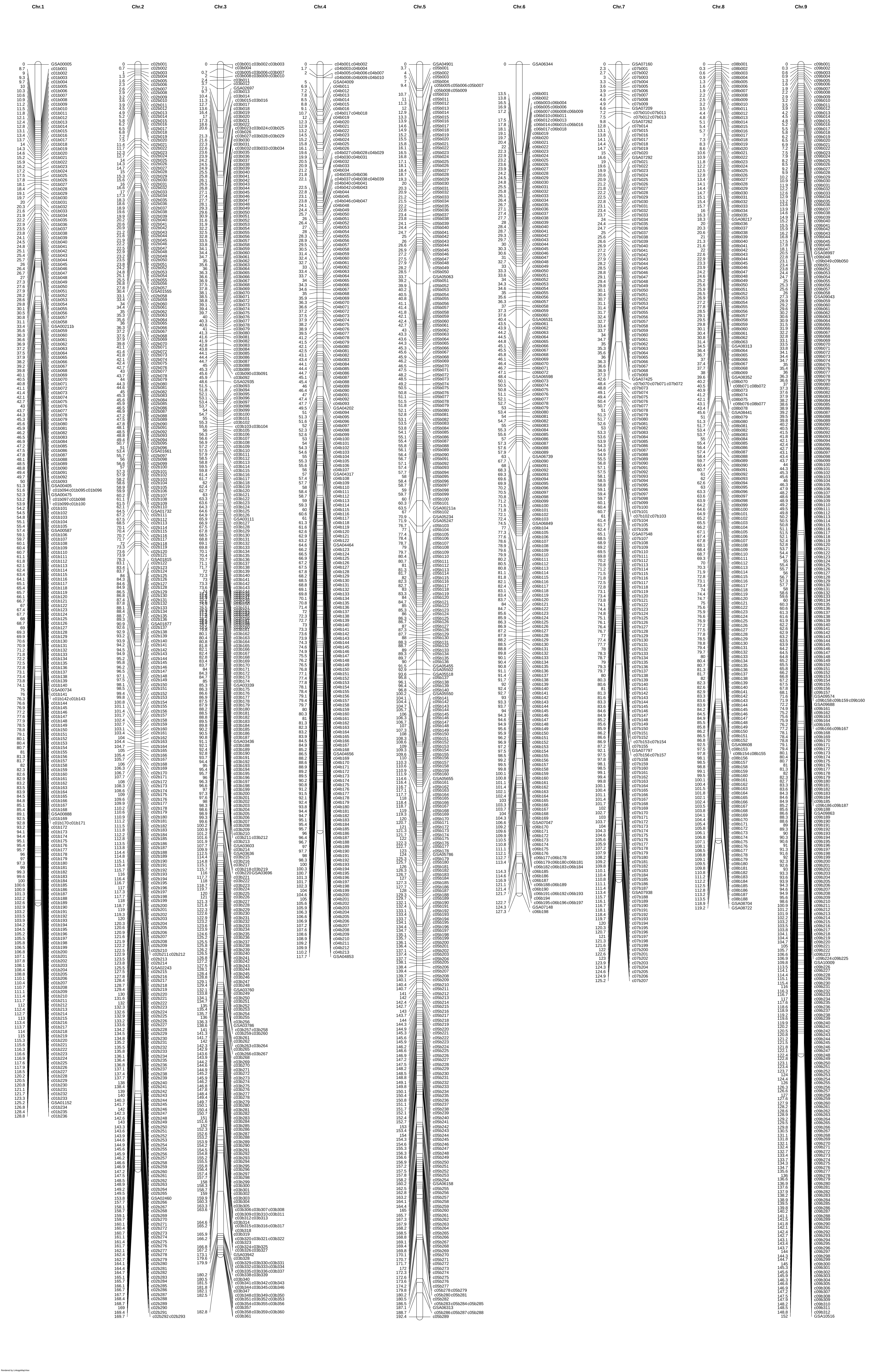
Chr.1
Chr.2
Chr.3
Chr.4
Chr.5
Chr.6
Chr.7
Chr.8
Chr.9
Rendered by LinkageMapView
